# The Single T65S Mutation Generates Brighter Cyan Fluorescent Proteins with Increased Photostability and pH Insensitivity

**DOI:** 10.1371/journal.pone.0049149

**Published:** 2012-11-02

**Authors:** Asma Fredj, Hélène Pasquier, Isabelle Demachy, Gabriella Jonasson, Bernard Levy, Valérie Derrien, Yasmina Bousmah, Gallia Manoussaris, Frank Wien, Jacqueline Ridard, Marie Erard, Fabienne Merola

**Affiliations:** 1 Laboratoire de Chimie Physique, CNRS, Université Paris Sud, Orsay, France; 2 Synchrotron SOLEIL, L'Orme des Merisiers, Gif sur Yvette, France; Stanford, United States of America

## Abstract

Cyan fluorescent proteins (CFP) derived from *Aequorea victoria* GFP, carrying a tryptophan-based chromophore, are widely used as FRET donors in live cell fluorescence imaging experiments. Recently, several CFP variants with near-ultimate photophysical performances were obtained through a mix of site-directed and large scale random mutagenesis. To understand the structural bases of these improvements, we have studied more specifically the consequences of the single-site T65S mutation. We find that all CFP variants carrying the T65S mutation not only display an increased fluorescence quantum yield and a simpler fluorescence emission decay, but also show an improved pH stability and strongly reduced reversible photoswitching reactions. Most prominently, the Cerulean-T65S variant reaches performances nearly equivalent to those of mTurquoise, with QY  = 0.84, an almost pure single exponential fluorescence decay and an outstanding stability in the acid pH range (pK_1/2_ = 3.6). From the detailed examination of crystallographic structures of different CFPs and GFPs, we conclude that these improvements stem from a shift in the thermodynamic balance between two well defined configurations of the residue 65 hydroxyl. These two configurations differ in their relative stabilization of a rigid chromophore, as well as in relaying the effects of Glu222 protonation at acid pHs. Our results suggest a simple method to greatly improve numerous FRET reporters used in cell imaging, and bring novel insights into the general structure-photophysics relationships of fluorescent proteins.

## Introduction

Green fluorescent proteins (GFPs) allow the construction of highly integrated, targeted and evolutive optical biosensors that have profoundly renewed our understanding of the cellular machinery. In particular, GFP spectral variants combined with Förster resonant energy transfer techniques (FRET) allow the specific imaging of chemical activities and molecular interactions inside living cells [1]–[4]. In most cases, a cyan fluorescent protein (CFP) derived from *Aequorea victoria* GFP (*Av*GFP) is used as the donor, in combination with a yellow acceptor [5], [6], and these two types of spectral variants are the subject of intense genetic engineering efforts. The original cyan fluorescent protein was obtained by Tsien and coworkers by substituting a tryptophan residue for the central tyrosine of the *Av*GFP chromophore [7], a mutation that required further mutagenesis to restore sufficient fluorescence emission [8], [9]. Unfortunately, ECFP still suffers from a relatively low fluorescence quantum yield (QY = 0.40), a limited photostability and a complex, pH sensitive fluorescence emission [10]. These drawbacks are major limiting factors in the design of sensitive and quantitative FRET protocols, especially when using fluorescence lifetime imaging detection (FLIM). These problems have been only partially alleviated in Cerulean, carrying three more mutations (S72A/Y145A/H148D), which resulted in an increased quantum yield and a somewhat simpler fluorescence decay [11], but a decreased photostability [12].

While ECFP and Cerulean are now incorporated in the vast majority of genetically encoded FRET constructs, they have been subjected to further engineering. This has led recently to mTurquoise [13], quickly followed by mCerulean3 [14] and mTurquoise2 [15], which now reach very impressive and near ultimate photophysical performances (QYs close to 0.9 and single exponential emission decays). Besides substantial amino-acid differences, due to the large scale random mutagenesis approaches used in their development, these improved CFP versions have only three common mutated positions (Thr65, Ser72 and His148), as compared to ECFP. Assessing the effective role of these mutations in their improved performances would provide invaluable lessons about the general structure-photophysics relationships in these proteins, and would also help in making the best choices in the future design of FRET constructs. After our study of the single point mutant ECFP-H148D [10], we report here on the detailed effects of the T65S mutation, in the different contexts of ECFP, Cerulean and mTurquoise.

The T65S mutation reverts one of the very first mutations ever introduced in *Av*GFP, inherited by many of its popular variants currently used in biological imaging [9]. The original S65T mutation was accompanied by some decrease in the quantum yield of *Av*GFP and several of its variants [8], [9], but in the case of tyrosine-based chromophores, this was compensated by a higher proportion of the anionic, fluorescent form of the chromophore. The reverse T65S mutation in the context of a cyan fluorescent protein was selected during the development of mTurquoise, through a large-scale, fluorescence lifetime screening of the randomized residue 65 codon [13]. The same mutation was subsequently introduced by site-directed mutagenesis during the development of mCerulean3 [14], and appeared again as a very effective step towards improved fluorescence quantum yields. Therefore, we decided to evaluate its consequences in the ECFP and Cerulean ancestors.

We find that restoring a serine residue at position 65 of CFP variants results in a whole series of drastic changes in their fluorescence properties. Not only does it increase the fluorescence quantum yield and reduce the heterogeneity of fluorescence emission, but it also markedly reduces the pH sensitivity, inhibits reversible photoreactions, and delays irreversible photobleaching. To understand how the simple removal of a methyl group can lead to such profound photophysical changes, we analyzed the X-ray crystallographic structures of several CFP and GFP variants. We found that the residue 65 hydroxyl in CFPs as well as GFPs can exist in two different conformations, characterized by a very different H-bonding status. While a serine hydroxyl preferably forms a stable H-bond with an important nearby water layer, likely favoring a rigid and fluorescent chromophore, a threonine residue can also engage in different, possibly destabilizing interactions with the imidazolinone moiety of the chromophore or the local α-helix backbone. While our study clearly demonstrates the multiple benefits of the T65S mutation for the amelioration of cyan fluorescent proteins, it also provides a consistent structural rationale for its beneficial consequences, that may guide the future genetic engineering of many other GFP variants.

## Materials and Methods

### Materials and molecular cloning

2-[N-Morpholino] ethane sulfonic (MES; Sigma), CAPS (Sigma), Bis-tris propane (Sigma) and citric acid (Sigma-Aldrich) buffers as well as H_2_SO_4_ (Sigma-Aldrich) and NaOH (Aldrich) were used as received.

### Mutagenesis

The bacterial expression vector pHis-CFP for the His-tagged enhanced GFP variant ECFP (AvGFP F64L/S65T/Y66W/N146I/M153T/V163A/H231L in pProEx HTa) is a generous gift from Dr. R. Grailhe from Institut Pasteur Korea. The T65S, S72A, Y145A, H148D, S175G or A206K mutation were sequentially introduced in pHis-CFP and pECFP-N1 using the QuickChange Site-Directed Mutagenesis Kit from Stratagene and following primers, where the mutagenic codons are bold:

T65S: 5′-CGTGACCACCCT**GAG**CTGGGGCGTGCAGTGC-3′

5′-GCACTGCACGCCCCAG**CTC**AGGGTGGTCACG-3′

S72A: 5′-CGTGCAGTGCTTC**GCC**CGCTACCCCGACCAC-3′

5′-GTGGTCGGGGTAGCG**GGC**GAAGCACTGCACG-3′

Y145A: 5′-GCTGGAGTACAAC**GCC**ATCAGCGACAACGTC-3′

5′-GACGTTGTCGCTGAT**GGC**GTTGTACTCCAGC-3′

H148D: 5′-CAACTACATCAGC**GAC**AACGTCTATATCACC-3′

5′- GGTGATATAGACGTT**GTC**GCTGATGTAGTTG -3′

S175G: 5′- CAACATCGAGGACGGC**GGC**GTGCAGCTCGCC -3′

5′- GGCGAGCTGCAC**GCC**GCCGT CCTCGATGTTG -3′

A206K: 5′- CCTGAGCACCCAGTCC**AAG**CTGAGCAAAGACCCC -3′

5′- GGGGTCTTTGCTCAG**CTT**GGACTGGGTGCTCAGG- 3′.

The mutations were checked by DNA sequencing. The amino-acid composition of ECFP, Cerulean and mTurquoise are respectively:

ECFP *Av*GFP-F64L, S65T, Y66W, N146I, M153T, V163A, H231L.

Cerulean ECFP-S72A, Y145A, H148D.

mTurquoise ECFP-T65S, S72A, H148D, S175G, A206K.

### Purification of the ECFP derivatives

Production and purification of His-tagged recombinant CFP variants was performed using TOP10 bacterial cells as described previously [16]. Competent cells were transformed with the pHis-CFP vector. A volume of 1.5 L of Luria–Bertani (LB) medium containing ampicillin (100 µg mL^−1^) was inoculated with a 25 mL starter culture that was grown overnight. Protein production was induced (OD_600_≈0.6) using isopropyl-β-D-thio-galactopyranoside (IPTG, 1 mM). After 18 h of culture at 30°C, bacteria were harvested by centrifugation and frozen. The cells were resuspended in lysis buffer (30 mL; 50 mM Tris–HCl, 5 mM 2-mercaptoethanol, 1 mM phenylmethylsulphonyl fluoride and 0.02 µg mL^−1^ DNase), and sonicated. The cell debris were removed by centrifugation. The solution was then applied to a column containing nickel-nitriloacetic acid (Ni–NTA) agarose (Sigma) for 1 h. The column was then washed and the protein was eluted (30 mM NaH_2_PO_4_, 100 mM NaCl and 150 mM imidazole, pH 7.5). The purified fusion protein His-CFP was further concentrated and the elution buffer was replaced by a pH buffer (2 mM CAPS-MES-Bis-tris propane or 2 mM citric acid buffer, pH 7.4). Protein concentration was quantified using a home-made Bicinchronic acid assay with BSA as standard. Due to uncertainties in protein assays, all extinction coefficients reported in this study are within 10–15% error. SDS-PAGE of the purified protein shows a single band corresponding to 29 kD and sample purity was assessed to be superior to 95%.

Data reported in the present work were obtained on proteins carrying the polyhistidine tag used for purification.

### Absorption and fluorescence spectroscopy

All fluorescence measurements were performed at 20°C±0.1°C, using 3 mm path length quartz cuvettes with black side walls (Hellma 105-251-QS, Hellma Ltd). Buffer solutions contained 30 mM CAPS, 30 mM MES and 30 mM Bis-tris propane at pHs ranging from 11 to 5.5, and 50 mM citric acid at pHs ranging from 5.5 to 2.5. Aliquots from a concentrated protein solution were diluted into buffers previously adjusted to the appropriate pHs at 20°C by addition of H_2_SO_4_ or NaOH (measurement accuracy:0.1 units), avoiding direct addition of concentrated acid into ECFP solutions. The final protein concentration was typically 10 µM for steady state and time resolved studies.

#### Steady state absorption and fluorescence

UV-Visible spectra were performed on a Perkin Elmer Lambda 900 spectrophotometer. The extinction coefficients of all proteins were determined by the Lambert Beer's law using the protein concentrations estimated from the BCA assay. The steady state emission and excitation spectra were recorded on a Spex Fluorolog 1681 spectrofluorimeter. Band widths of 1 nm for excitation and emission were used. Spectra were collected with integration set to 1 s and increment 1 nm. In all experiments, signal from pure buffer was subtracted as a background. The maximal average power at the sample was 40±2 µW, with a beam section about 0.15 cm^2^. Fluorescence quantum yields were estimated using ECFP as a standard (QY = 0.40) [17].

#### Time resolved fluorescence

The fluorescence decay curves were recorded using the time-correlated single photon counting technique (TCSPC) as described in [10]. The excitation source was a pulse-picked, frequency doubled, mode-locked Ti:Sapphire laser (MIRA 900, Coherent, Watford, UK) and the excitation wavelength was 420 nm. The average laser power at the sample was typically 1–1.4 µW (beam waist about 1−2 mm). The full width at half maximum of the instrumental response function (IRF) was typically 60–70 ps. The emission monochromator bandpath was 6 nm for most experiments and typically 15–25×10^6^ total counts were collected in each decay curve.

Photoreactions were carefully controlled along steady-state and time-resolved fluorescence spectroscopy experiments. The excitation densities used were in all cases below 10^−3^ W/cm^2^, while the collection of fluorescence decays was combined to real-time monitoring of the average decay time position and intensity of the fluorescence along data acquisition. Any experiment showing more than 10% intensity loss or 50 ps time-drift over the complete data acquisition were discarded.

### Data analysis

Each experimental fluorescence decay curve F(t) was analyzed individually together with its IRF using the maximum entropy method as described in [10], [18], [19]. This analysis assumes that the experimental decay F(t) is the convolution product:
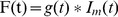
(1)where *g(t)* is the measured IRF, and *I_m_(t)* is the pure fluorescence decay law for instant excitation. The analysis assumes that the fluorescence decay law is composed of a large number of exponential terms. The total decay is then given by:
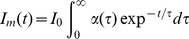
(2)where α(τ) is the distribution of normalized pre-exponential amplitudes (i.e. ∫ α(τ)dτ  = 1), and I0 is an arbitrary factor incorporating the instrumental conditions of the measurement. A time-shift between F(t) and g(t) was also optimized, and the reduced χ2 was in the range of 0.97 to 1.05 in all cases, with randomly distributed residuals and autocorrelation functions.

From the fluorescence lifetime distribution *α(τ)* recovered by this method, a small number of individual components *τ_i_* and their corresponding pre-exponential amplitudes *(a_i_)* are obtained by partial integration over each separate peak observable in the distribution. The distribution *α(τ)* is used for computing the average fluorescence lifetime <*τ _f_*>, defined as the amplitude-averaged decay time, which should follow a proportionality relationship to the fluorescence quantum yield [10], [20]:
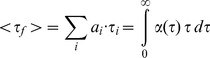
(3)


The experimental errors on *a_i_, τ_i_* and <*τ _f_*> were obtained from the standard deviations over repeated identical experiments.

### Synchrotron radiation circular dichroism

Measurements were carried out on the DISCO beamline [21] at Soleil synchrotron (Gif sur Yvette, France). Calcium Fluoride circular cuvettes (Hellma)[22] of 11 μm were used. Protein concentrations were typically 8 mg/L at pH 2.5 and 18 mg/L at pH 7.4. All samples were equilibrated overnight against their buffer (30 mM CAPS, 30 mM MES and 30 mM Bis-tris propane at pH 7.4 and 30 mM citric acid at pH 2.5). Spectra for each protein were obtained by averaging 3 scans, from 170 to 280 nm with 1 nm intervals per second. Three consecutive scans of the baseline (using the buffer) were obtained in the same manner and averaged. For all proteins, experiments were recorded at 25°C. Spectra of buffer were subtracted from those of corresponding samples. The 260–270 nm region was set to zero, and the resulting spectra were calibrated with CSA (D-10-camphorsulfonic acid) using the CDtool software [23]. The units of molar differential extinction coefficient are M^−1^ cm^−1^ [24]. The secondary structure determination was performed on DICHROWEB [25] using the CDSSTR and CONTINLL algorithms and the SP175 reference datasets [26]. Both algorithms give similar results. Here are presented results obtained from the CDSSTR analysis program. The NRMSD fit parameter ranges from 0.030 to 0.050 for all proteins.

### Photobleaching experiments

#### CFP labeling of agarose beads

Nickel loaded agarose beads (Sigma) were labeled with recombinant His-tagged CFP proteins. 100 µL of sedimented beads, previously washed and equilibrated with phosphate buffer (pH 7.5), were incubated with 1–5 µM of purified CFP protein in a total volume of 1 mL during 1h under gentle shaking in the cold. The beads were then centrifuged 5 min at 5000 rpm, washed twice and finally resuspended in PBS buffer. A few µL of the bead suspension were deposited onto a 25 mm Ø microscope coverslip for bleaching experiments.

#### Expression of cytosolic CFPs

MDCK cells cultivated on 25 mm Ø coverslips were transiently transfected with the eukaryotic expression plasmids of the different CFP variants using Lipofectamine 2000 following the manufacturer recommendations (Invitrogen) and were studied 24 to 48 hours after transfection.

#### Irradiation and imaging conditions

CFP fluorescence photobleaching experiments were performed at 20.0±0.5 °C on a wide field epi-fluorescence microscope equipped with a x60, 1.2NA water immersion objective (Nikon), an HBO 100W Hg lamp placed behind a fast shutter and a filter wheel, and using a CFP dichroic filter set for fluorescence detection (Omega XF114-2). The illumination power was adjusted by neutral density filters, the maximum power measured on the sample without any attenuation was approximately 200 µW (FieldMaster with Detector 13M41, Coherent), and the radius of the illuminated field was estimated as 185 µm, leading to an average irradiance on the sample of about 0.2 W/cm^2^. Homogeneity of illumination was better than 85% over the whole field of view. Single beads or groups of MDCK cells were placed at the centre of the imaging field and the fluorescence intensity imaged by a cooled CCD camera (ORCA-AG Hamamatsu) was quantified using the NIH ImageJ package. We found no significant dependence of the measured photobleaching rates on the bead size, nor on the bead labeling density.

## Results

### The T65S mutation improves the homogeneity and quantum yield of CFP fluorescence

**Figure 1:**
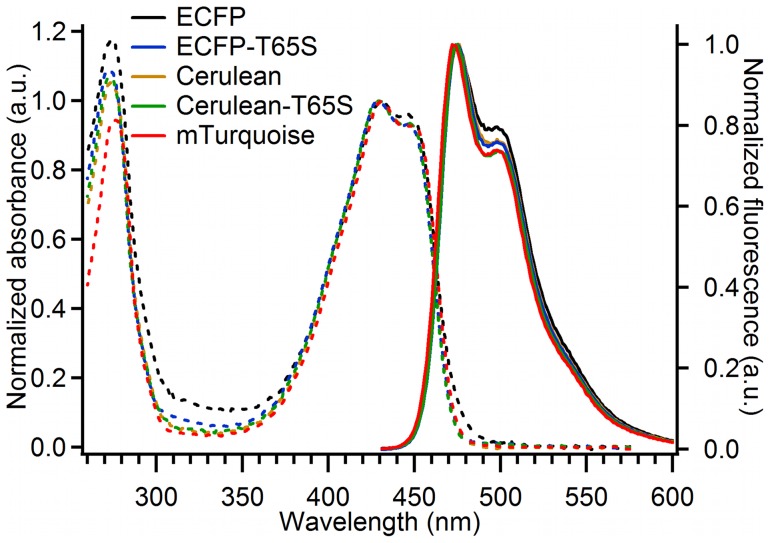
Spectral properties of purified CFP variants at neutral pH. Absorption (dashed lines) and emission (solid lines) spectra were normalized to maximum of the chromophore band. Emission spectra were recorded with excitation at 420 nm.

We first compared the absorption and fluorescence properties of purified ECFP, Cerulean and mTurquoise, to those of the single point mutants ECFP-T65S and Cerulean-T65S, at neutral pH and room temperature. The absorption and fluorescence spectra of these CFP variants are highly similar, and display the typical double-hump of indole-based chromophore bands, with two absorption maxima at 430 nm and 445 nm, and two emission maxima at 474 nm and 500 nm (Figure 1). Taking into account experimental uncertainties, we also obtain very similar absorption coefficients for all studied variants, in the range of 32000±4000 M^−1^cm^−1^ at 430 nm (Table 1).

**Table 1:** Photophysical properties of cyan fluorescent proteins.

					Photostability^a^			
					on agarose beads			in living cells
Protein	ε (10^3^ M^−1^ cm^−1^)	Quantum Yield	Average lifetime (ns)	pK_1/2_	% Rev^b^	τ_Rev_ (s)^c^	τ_Irrev_ (s)^d^	τ_Irrev_ (s)^d^
			**±0.04 ns**		**±2%**	**±0.1s**	**±5%**	**± SD**
**ECFP**	29.0	0.40^e^	2.52	5.6	23	0.6	700	726±88 (N = 37)
**ECFP-T65S**	28.5	0.59	3.30	4.5	3	1.0	940	828±89 (N = 23)
**Cerulean**	29.2	0.67	3.05	5.2	33	1.0	610	643±97 (N = 21)
**Cerulean-T65S**	34.8	0.84	3.96	3.6	3	0.8	772	ND
**mTurquoise**	36.2	0.85	4.06	3.4	<1	1.4	1245	1482±182 (N = 29)

a All parameters determined as described in Text S1.

b Relative initial decrease in fluorescence intensity after sudden illumination at 0.2 W/cm^2^.

c Exponential time constant of the initial fluorescence intensity decrease.

d Exponential time constant of the irreversible loss in fluorescence under prolonged illumination at 0.2 W/cm^2^.

e from [17].

ND Not Determined.

**Figure 2:**
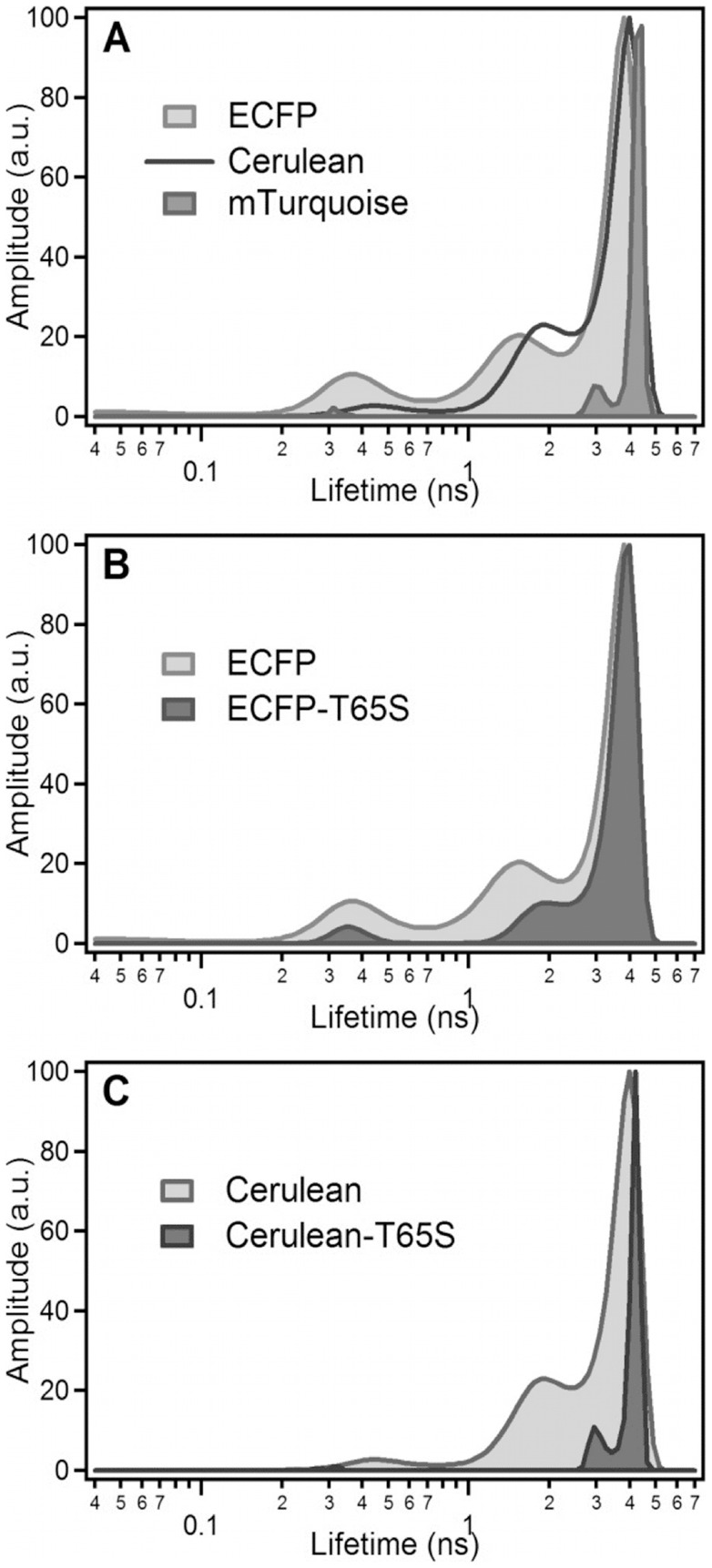
Fluorescence lifetime distributions of purified CFP variants at neutral pH. The distributions are obtained from maximum entropy analysis of the fluorescence decay curves of (A) ECFP, Cerulean and mTurquoise, (B) ECFP and ECFP-T65S and (C) Cerulean and Cerulean-T65S. For each protein, the distribution shown is an average of six independent experiments recorded at pH 7.4 and T = 20°C.

Despite close spectral similarity, these CFP variants markedly differ in their fluorescence quantum yields and average fluorescence lifetimes (Table 1), as well as in the pattern of their fluorescence lifetime distributions, as recovered from their fluorescence emission decays (Figure 2). These lifetime distributions comprise in all cases a major, long fluorescence lifetime, associated to variable amounts of short components (Table S1). We have already reported on the complex photophysics of ECFP [10], with a lifetime distribution including about 50% of multiple short components (Figure 2A). Cerulean has an increased quantum yield as compared to ECFP, but retains a heterogeneous fluorescence emission, its longest lifetime contributing to only 64% of the total decay amplitude (Figure 2A and Table S1). By contrast, mTurquoise has both a high quantum yield (Table 1) and a considerably simplified emission decay, with now 87% of a long lifetime component (Figure 2A).

The T65S single mutation brings major improvements to the performances of the original ECFP and Cerulean forms. The fluorescence quantum yield of ECFP-T65S is increased by 48% compared to ECFP, while that of Cerulean-T65S increases by 25% compared to Cerulean (Table 1). The T65S mutation also results in a marked simplification of the fluorescence lifetime distributions (Figure 2B,C). In the case of Cerulean, the T65S mutation leads to a fluorescence quantum yield and average lifetime very close to those of mTurquoise (Table 1), and similarly to mTurquoise, the fluorescence decay follows a near-single exponential kinetics dominated by 83% of a long lifetime (Figure 2C).

### The T65S mutation improves the pH stability of CFP fluorescence in the acid range

**Figure 3:**
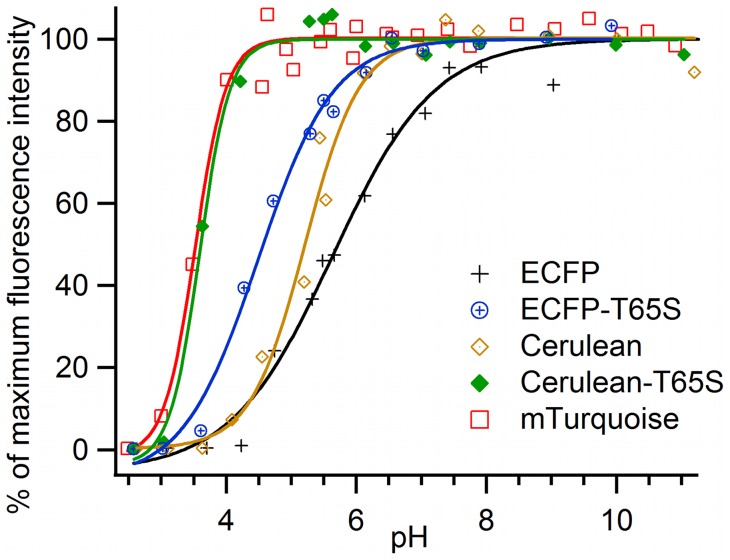
pH dependence of the fluorescence intensity of purified CFP variants. Fluorescence intensities were excited at 420 nm and detected at 474 nm (Δλ = 6 nm). Solid lines correspond to the best fits to a sigmoidal analytical model. Experimental data were normalized to 100% maximum of their analytical fits.

**Figure 4:**
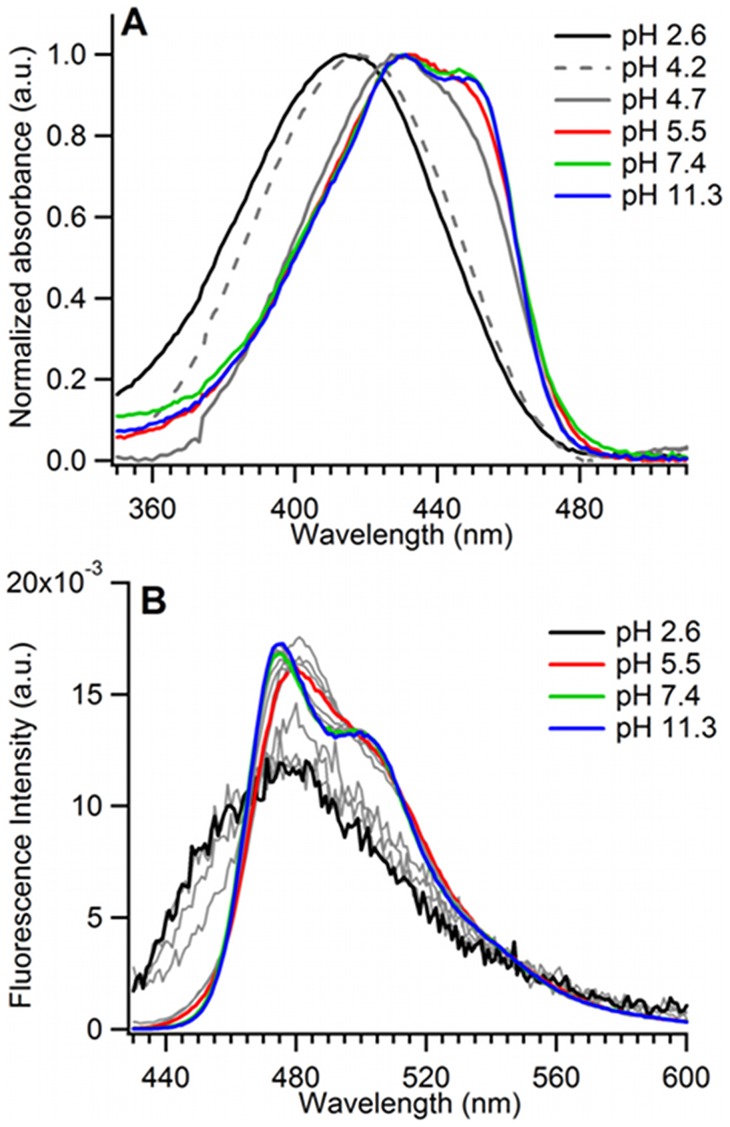
pH dependence of the spectral properties of purified ECFP. (**A**) Absorption spectra normalized to unit maximum absorbance, and (**B**) emission spectra normalized to unit surface. The spectra corresponding to 50% fluorescence intensity loss (pH = pK_1/2_) is represented by a continuous red line. The absorption spectrum represented by a dashed gray line corresponds to the first acid pH at which a typical denaturated spectrum is observed.

**Figure 5:**
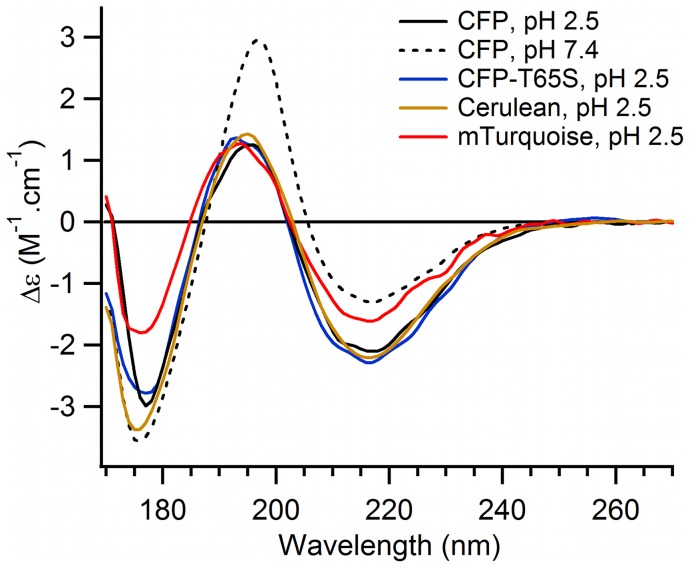
Synchrotron radiation circular dichroism on purified CFP variants at neutral and acid pHs. Comparison of the SRCD spectra of ECFP at pH 7.4 (dashed line) and different CFP variants at pH 2.5 (solid lines). The SRCD spectra were recorded at 25°C.

While very little spectroscopic perturbations are observed when going from neutral pH up to pH 11, all studied CFP variants undergo a complete loss in fluorescence intensity when the pH is decreased down to pH 2.5 (Figure 3). At pH 2.6, the absorption and fluorescence spectra of ECFP are broad and structureless (Figure 4), with an absorption maximum shifted to 414 nm (ε_414nm_ = 34000 M^−1^ cm^−1^), and a fluorescence quantum yield lower than 3 10^−3^. All other CFP variants display very similar, structureless and blue shifted spectra at acid pH (Figure S1 and Figure S2), while their CD spectra in the 170 nm–270 nm range indicate major changes in secondary structure, as expected for a denaturation (Figure 5 and Figure S3).

The shape and position of the acid induced loss of fluorescence varies however markedly between the different CFP variants (Figure 3). The half transition points for ECFP (pK_1/2_ = 5.6) and Cerulean (pK_1/2_ = 5.2) lie well above pH 5, indicating a poor pH stability. The single mutation T65S markedly improves this stability, shifting the pK_1/2_ of ECFP-T65S and Cerulean-T65S by 1.1 and 1.6 pH units respectively. Cerulean-T65S, with a half transition point near 3.6, achieves an overall pH stability equivalent to that of mTurquoise. Together with mCerulean3 (pK_1/2_ = 3.2) [14] and mTurquoise2 (pK_1/2_ = 3.1) [15], all carrying the T65S mutation, these improved CFP variants actually rank among the most pH-stable of the GFP literature [12], [27].

The onset of acid-induced perturbations of CFP fluorescence, close to physiological pHs, is particularly important for live cell imaging. In the case of ECFP, the fluorescence starts to decrease close to pH 7, and the decrease extends over more than 3 pH units. Actually, the ECFP transition is much broader than predicted for a single protonation scheme described by the Henderson-Hasselbalch relationship, which suggests a complex sequence of events. For Cerulean, the transition is well fitted by a single protonation model. By contrast, the transition is steeper than expected for a single protonation event for mTurquoise and Cerulean-T65S, as is frequently observed in the case of the highly cooperative, acid-induced unfolding of globular proteins [28].

**Figure 6:**
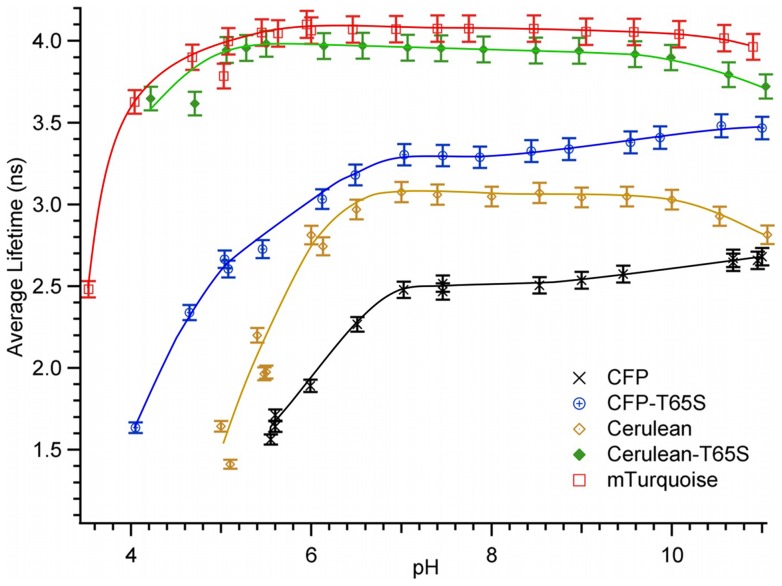
pH dependence of the average fluorescence lifetime of purified CFP variants. Average lifetimes were determined at 20°C from integration of the corresponding lifetime distributions. Solid lines are for eye guidance only.

The pH-induced fluorescence intensity changes are paralleled with changes in the corresponding average fluorescence lifetimes (Figure 6). We have already reported on the pH dependence of the ECFP fluorescence decays [10]. For all CFP variants, in line with their corresponding loss in fluorescence quantum yield, the average fluorescence lifetime decreases rapidly at acid pH. However, the lifetime decreases appreciably slower than the quantum yield, probably indicating a growing proportion of non fluorescent forms: at pHs corresponding to 50% intensity loss, the average fluorescence lifetime has decreased by only 32%, for ECFP and ECFP-T65S and by 36% for Cerulean (Cerulean-T65S and mTurquoise do not allow a comparable analysis due to limited data and steepness of their transition). Towards basic pHs, the measurement accuracy of average fluorescence lifetimes reveals more subtle perturbations: variants carrying a histidine at position 148 (ECFP and ECFP-T65S) undergo an increase in fluorescence lifetime, while variants carrying an aspartate (Cerulean, Cerulean-T65S and mTurquoise) undergo on the contrary a decrease (Figure 6).

### The T65S mutation suppresses detectable intermediates along the acid transition

The detailed analysis of absorption and fluorescence spectra of CFP variants along the acid transition provides multiple evidences for the build up of intermediate forms (Text S1). Notably, in the case of ECFP, a transient red shift of the maximum of fluorescence emission is observed at pH 5.5, that continues down to pH 4.7 (Figure 4B). When the pH is further decreased, the emission maximum shifts back to the blue. This behaviour cannot be accounted for by a simple two-state transition (Figure S2F), and provides evidence for an intermediate, red-shifted acid form of ECFP clearly distinct from both its neutral and acid-denatured states. This red-shifted intermediate vanishes when the T65S mutation is introduced into ECFP (Figure S2C). Similarly, in the case of Cerulean, there is evidence below the pK_1/2_ for yet a different spectral intermediate, characterized by a marked shoulder in the blue in both absorption and fluorescence (Figure S1B and Figure S2B). This blue-shifted form of Cerulean at acid pH has already been reported [29], and was ascribed to chromophore *cis trans* isomerization, favored by the protonation of Asp148 [29], [30]. Again, this intermediate isomerized state becomes undetectable in Cerulean-T65S (Figures S1D and S2D). By contrast, the absorption and fluorescence spectra of Cerulean-T65S, as well as those of mTurquoise (Figures S1E and S2E), are always well described by mixtures of their neutral and acid forms (Figures S1F and S2F), with no evidence for intermediate spectral perturbations. In these two variants, the loss of fluorescence intensity, the loss of two-hump structure, and the blue expansion of the spectra take place nearly simultaneously in a very narrow pH range.

### Reversible photoreactions of ECFP attached to agarose beads

**Figure 7:**
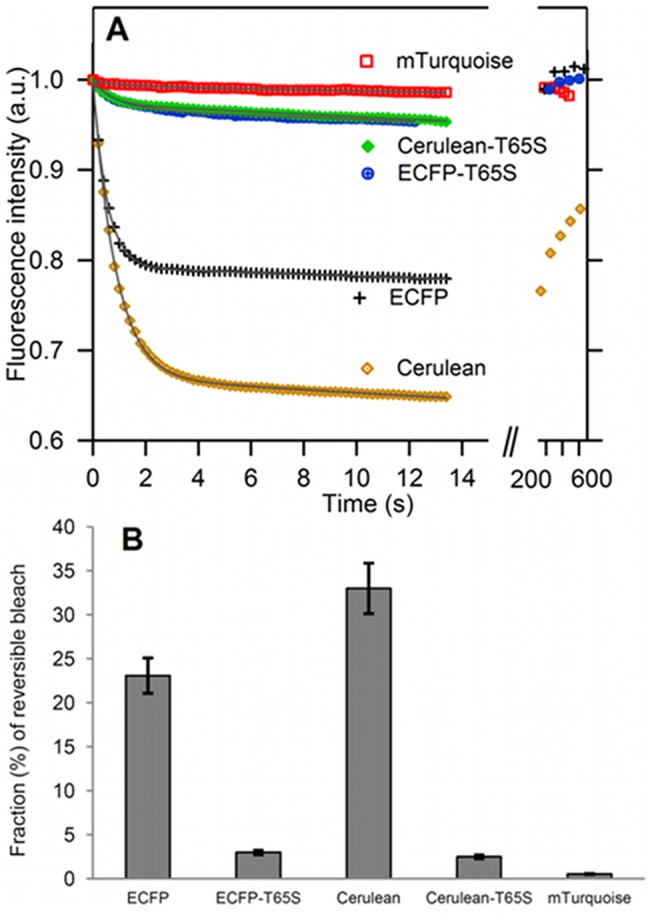
Reversible photobleaching of purified CFP variants. (A) Reversible bleaching kinetics performed on agarose beads labeled with purified CFPs. After prior equilibration in the dark, sudden and constant illumination at 0.2 W/cm2 was applied for less than 15 sec, while camera images were taken every 200 ms. Illumination was then stopped, and, after a minimum of 3 min in the dark, a short series of fluorescence images was collected to check for reversibility. Continuous lines are best fits to the model F^norm^ =  y_0_+y_1_t+y_2_ exp(−t/τ_Rev_) (see Text S1). (B) Amplitudes of the reversible photobleaching responses of the CFP variants.

Many natural or genetically engineered fluorescent proteins from various sources have been shown to undergo reversible light induced conversions between optically distinct states. These fluorescent proteins, commonly referred to as reversibly switchable fluorescent proteins (RSFPs), have found useful applications in pulse chase experiments or super resolution optical microscopy [12], [31]. It is increasingly recognized that similar reversible photoreactions are, to various extents, common to most fluorescent proteins, and that this may have major consequences for their use in biological imaging [14], [32], [33]: in standard FRET applications, the fluorophores should be, as much as possible, devoid of such uncontrolled photoreactions. However, quantitative data on the reversible photoswitching properties of cyan and yellow fluorescent proteins remain quite sparse. These reactions are most easily monitored on purified, immobilized fluorescent proteins, eliminating interferences by Brownian diffusion or living cell movements. In line with earlier reports [14], [32], we find that purified ECFP bound to agarose beads undergoes a pronounced and reversible transient bleaching under irradiation in its chromophore absorption band. Under sudden wide-field illumination using maximum Hg lamp power, the fluorescence intensity drops exponentially by 23%, with a time constant of less than one second (Figure 7, Table 1). Following this transient response, there is a slower decrease in fluorescence intensity of about 0.1% per second, most likely due to irreversible photobleaching (see below). If the illumination time is kept sufficiently short, and after several minutes in complete dark, the fluorescence intensity reverts back to its initial level (Figure 7A).

Moreover, in agreement with Sinnecker and coworkers, we find that the recovery of ECFP fluorescence after transient bleaching is accelerated by moderate illumination at the same wavelengths (Figure S4). This shows that the return of ECFP back to its fluorescent state is also light-activated. As a consequence, the stationary level of fluorescence reached after a few seconds of illumination must correspond to a steady state regime, where both “off” and “on” photoreactions take place at equal rates. A minimum two-state kinetic model can be used to describe this system (Text S1, Figure S5 Figure S6 and Figure S7). This model is characterized by the two rate constants k_off_ and k_on_, which describe the elementary reactions of reversible bleaching and photoactivated return respectively. According to this model, the apparent relaxation time τ_rev_ in our bleaching experiments is the inverse sum of the two rate constants (k_on_+k_off_)^−1^, while the steady-state level of fluorescence reached after a few seconds is given by k_on_/(k_on_+k_off_), from which the elementary rate constants k_on_ and k_off_ can be obtained (Table S2). From these rate constants, one can also derive estimates of the photoconversion quantum efficiencies in both directions (Text S1): the obtained values, Φ_off_  = 1% and Φ_on_  = 6%, show that ECFP is indeed a very efficient RSFP [31], [33]–[35].

### The T65S mutation markedly decreases the rate and amplitude of reversible CFP photobleaching

We have studied the transient bleaching of other CFP variants according to the same experimental procedure. Upon sudden illumination, all CFPs undergo reversible fluorescence drops on similar time scales of a few seconds, but with widely different amplitudes (Figure 7). We find that Cerulean displays a pronounced 33% decrease, while the response of mTurquoise, below 1%, is barely detectable (Figure 7B). Most remarkably, the T65S mutation introduced in either ECFP or Cerulean markedly reduces the amplitude of the transient decrease, to less than 3% in both cases (Figure 7B).

Comparing the elementary rate constants k_on_ and k_off_ for each CFP variant provides further insights on the photoswitching mechanisms (Table S2). First, the pronounced transient photoresponse of Cerulean results from a relatively low value of k_on_, the rate of photoactivated *return* to the fluorescent state (Table S2). Strikingly, Cerulean displays also a slower fluorescence recovery in the dark, as compared to the other variants (Figure 7A). Therefore, the lack of photostability of Cerulean is mostly due to a slower back reaction to the fluorescent state, whether it takes place in the excited state, as a photoactivated reaction, or in the ground state, as a thermally activated reaction. By contrast, ECFP-T65S and Cerulean-T65S display a 10 fold decrease in their rate of reversible bleaching k_off_, with only moderate changes in photoactivated return, as compared to ECFP and Cerulean (Table S2). This trend is even more pronounced in mTurquoise, which has a k_off_ rate constant 100-fold lower than ECFP. This largely compensates the relatively slow rate of photoactivated return k_on_ of mTurquoise (Table S2), resulting in near-undetectable photoresponses. Therefore, the major effect of the T65S mutation is to considerably slow down the elementary rate of reversible bleaching k_off_, leading to a reduced amplitude of transient bleaching.

The CFP photoreactions are readily observable also inside living MDCK cells expressing the different cytosolic CFP variants (Figure S8). However, using identical wide-field illumination conditions, the amplitudes of the responses appear markedly reduced as compared to *in vitro* conditions, while the relaxation times are significantly shorter (Table S2). A similar dampening of the apparent reversible photobleaching of cytosolic ECFP was noticed previously [32]. In the present case, this may stem in part from the limited time-resolution of our image acquisition set-up: if the photoreaction is significantly faster within the cell, a larger fraction of the fluorescence signal will be lost within the dead-time of the first image frame. Nevertheless, comparison of the different cytosolic CFP variants reveals very similar trends as compared to *in vitro* conditions: the T65S mutation strongly decreases the ECFP and Cerulean transient responses, Cerulean displays the most pronounced reversible photobleaching, and mTurquoise retains a nearly constant fluorescence signal over the observation time-window (Figure S8).

### The T65S mutation slows down irreversible CFP photobleaching

The irreversible bleaching reactions of the CFP variants were also studied. We used identical but prolonged wide-field illumination conditions on both immobilized agarose beads and cytosolic CFP variants. All variants display between 85% and 95% irreversible fluorescence loss after 30 min of irradiation at maximum Hg lamp power. The decay of intensity is approximately exponential (Figure S9), with less than 1% dark recovery and no detectable light-activated recovery, up to 10 min after switching off sample illumination. Experiments conducted on bead-immobilized and cytosolic proteins gave rather similar irreversible bleaching time constants (Table 1): we find in all cases that the single T65S mutation notably slows down the irreversible bleaching of Cerulean and ECFP, while mTurquoise displays a remarkable long term photostability (Table 1). In line with previous reports [12], Cerulean undergoes a somewhat faster bleaching than ECFP, showing that this variant has a decreased photostability both in terms of reversible and irreversible reactions.

## Discussion

### Excited state chromophore torsions behind the T65S effects

Restoring a wild-type serine instead of a threonine at position 65 in CFP variants has an astonishing variety of consequences: it simultaneously increases the fluorescence quantum yield and average lifetime, simplifies the emission kinetics, stabilizes the fluorescence against acid-induced perturbations, nearly suppresses reversible photobleaching, and slows down irreversible photobleaching. It is widely agreed today that excited-state torsion around their ethylenic central bridge is a common property of all GFP chromophores, that can lead to both dynamic fluorescence quenching and reversible photoisomerizations [36]. A better blockage of these excited state torsions likely accounts for the variety of observed effects of the T65S mutation in CFP variants. This idea is discussed in more detail below.

### The CFP fluorescence quantum yield is governed by local flexibility

Ultra-fast internal conversion due to excited state torsions is the major cause of the strong fluorescence quenching of model GFP chromophores in solution [37]. Inside well folded, globular fluorescent proteins, the chromophore is subjected to various degrees of conformational hindrance, and the extent to which these torsions can contribute to fluorescence quenching is difficult to assess. However, we have recently performed new types of molecular dynamic simulations of GFP-S65T in the excited state, showing that 90° torsions between the two aromatic moieties of the chromophore can take place on the nanosecond time scale inside the protein [38]. Such frequent torsions would significantly contribute to the decay of the excited state, and thus to fluorescence quenching. In addition, any perturbation or destabilization of the protein structure (for example, by detrimental mutations or acid pHs) that would only slightly increase the frequency of these torsions, will have direct measurable consequences on the fluorescence intensity.

There are multiple evidences that the ECFP protein is more flexible than its GFP parent [39]–[41], and this is thought to be the cause of its very complex fluorescence decays [10], [39]. Strikingly, we find that the brightest CFP forms mTurquoise and Cerulean-T65S are very close to follow single exponential fluorescence decays, similar to what is observed for their wild type parent *Av*GFP [10]. A single exponential fluorescence decay reflects a unique fluorophore environment on all times scales slower than the nanosecond. Conversely, a complex fluorescence emission kinetics reflects some local dynamic fluctuations of the fluorophore environment and/or excited state reactions of the chromophore itself. The simultaneous examination of the fluorescence lifetime distributions and quantum yields of the different CFP variants shows that any set of mutations that improves the CFP fluorescence quantum yield also decreases the fluorescence kinetic complexity. This correlation was verified for a large number of CFP mutants studied at the laboratory, and suggests that the CFP fluorescence quantum yield is directly governed by some type of local flexibility of either the chromophore and/or the surrounding protein matrix.

### Protein flexibility allows chromophore photoisomerization

Because of their flexibility, CFP variants might be also particularly more prone to chromophore photoisomerization. Due to excited-state torsions, all isolated synthetic GFP chromophores in solution undergo reversible photoinduced conversions between their different *cis trans* configurations [42], [43]. The photoconversion quantum efficiencies in both directions are very high for the CFP chromophore (Φ_cis_  = 20%, Φ_trans_  = 90%), while its different diastereoisomers have strongly overlapping absorption bands [42]. Similar *cis trans* chromophore photoisomerizations were shown to be involved in the photoswitching properties of nearly all RSFPs [44]–[48]. The efficiency of ECFP photoreactions, and the fact that the photoconverted CFPs retain a strong absorption in the normal chromophore band (allowing a very efficient photoactivated return to the “on” state), show that these reactions principally reflect, as for other RSFPs, a chromophore photoisomerization process.

### The T65S mutation stabilizes a more fluorescent CFP population

The T65S mutation would thus improve the fluorescence quantum yield and reduce the rate of chromophore photoisomerization, due to an increased local rigidity. More precisely, the T65S mutation slows down the on-> off photoisomerization reaction, with only moderate incidence on the reverse photoreaction rate. By contrast, the Cerulean case shows that other mutations leading to increased fluorescence, likely due as well to an increased local rigidity, may have instead a detrimental effect on the photostability, due to the concomitant slow down of fluorescence recovery reactions. In the case of T65S, the differential effect on the two rate constants would indicate a preferential, thermodynamic stabilization of the “on” fluorescent state, more than a purely dynamic effect associated to changes in activation barriers.

These stabilizing effects of T65S would in turn contribute to the marked increase in pH stability of variants carrying this mutation. At acid pHs, the fluorescence of mTurquoise and Cerulean-T65S appears only sensitive to some cooperative unfolding taking place below pH 4. In the case of ECFP and Cerulean, fluorescence perturbations begin close to physiological pHs, with multiple evidences, from this work and others [29], for protein-specific conformational intermediates that are distinct from, and clearly precede the final acid denaturation. These intermediates become undetectable when the single T65S mutation is introduced in both ECFP and Cerulean. Besides its direct action on chromophore torsions, the T65S mutation thus appears to contribute to a general stabilization of the native protein structure, that is able to counteract the consequences of early protonation events at acid pHs.

### Structural basis of the T65S mutation effects

The same T65S mutation simultaneously improved also the quantum yield, fluorescence homogeneity and pH stability of mTurquoise and mCerulean3, as compared to their respective SCFP3A and mCerulean2 precursors [13], [14]. Similar effects are observed in even more distant mutation contexts: in wild-type *Av*GFP, the S65T mutation led to a 19% decrease in fluorescence quantum yield [9], and a more complex fluorescence decay [49], while several blue variants of *Av*GFP with quite different chromophore structures were also found substantially more fluorescent when the S65T mutation was reverted [50], [51]. The general and extensive consequences of this mutation suggest a common, rather robust mechanism, that should have some clear structural grounds.

Very recently, the X-ray crystallographic structures of SCFP3A and mTurquoise have been published [15], providing the first opportunity to evaluate the structural correlates of the T65S mutation in the context of a cyan fluorescent protein. Not so unexpectedly however, their comparison is quite disappointing: in these two proteins, the local structure in the vicinity of the chromophore is highly similar (Figure S10). The only significant atomic displacements are those of the Leu220 side chain, a relatively remote and flexible group that is often modeled with multiple configurations [15], [39], [40]. Strictly speaking, as the computed root mean square distance between all heavy atoms of the chromophore cavity is only 0.17 Å (including Leu220 atoms) or even 0.08 Å (without the Leu220 atoms), the two structures should be considered identical, within the accuracy of X-ray crystallography. We found more significant, and previously unnoticed structural clues, through the comparative structural analysis of chromophore cavities amongst the whole *Av*GFP family.

**Figure 8:**
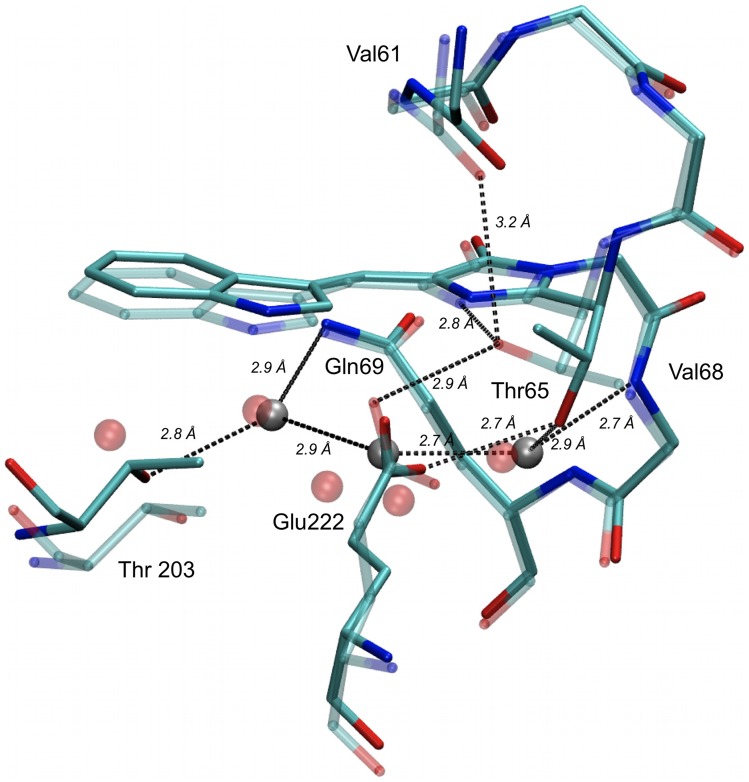
Alternate configurations of threonine 65 in the crystallographic structures of ECFP. Amino-acids and water molecules interacting with the residue 65 hydroxyl are shown, according to the structures of Lelimousin et al (2WSN, solid, grey) [40] and Bae et al (1OXD, transparent, red) [39]. Protein structures were aligned along their main chain backbones, water oxygens are shown as spheres and major H-bonds as dashed lines.

The configuration of the chromophore cavity in the crystallographic structures of ECFP and Cerulean [40] is very similar to that of SCFP3A and mTurquoise determined in the same laboratory [15]. However, the previously published structure of ECFP by Bae *et* *al* [39] and that of Cerulean by Malo *et* *al* [29] reveal significant differences (Figure 8 and Figure S11). While in the Lelimousin and Goedhart structures, the threonine 65 hydroxyl is H-bonded to a structural water molecule that lies just below the imidazolinone ring, in the Bae structure of ECFP (Figure 8), and in the Malo structure of Cerulean (Figure S11), the same hydroxyl points higher, in hydrogen bonding distance of the chromophore imidazolinone nitrogen, and of the Val61 carbonyl oxygen (Figure 8). We will name these two orientations of residue 65 the “Down” and “Up” configurations, respectively. Interestingly, we found very similar alternate configurations of the residue 65 hydroxyl in the crystallographic structures of *Av*GFP [52]–[55] and its single point mutant GFP-S65T [56]–[58]. Even more strikingly, all GFP proteins carrying a threonine at position 65 display an “Up” configuration, while all GFP proteins carrying the original serine 65 adopt the “Down” configuration (Text S1 and Figure S11).

Therefore, we conclude that the original “Down” configuration of the serine 65 hydroxyl in *Av*GFP is energetically disfavored when it is replaced by a threonine. Due either to its aliphatic or bulky properties, the extra methyl group of threonine 65 tends to be expelled from the polar surroundings of the chromophore, favoring the “Up” configuration. On the other hand, we assume also that the alternate occurrence of “Up” or “Down” configurations in different crystals of the same cyan fluorescent protein is one more evidence of their conformational plasticity. The different conditions used during crystal growth, handling and data acquisition (including possibly the crystallisation pH, see below) could select different, but unique final conformations. In solution at room temperature however, CFP variants will display variable proportions of both the “Down” and “Up” configurations, depending on their relative thermodynamic stability. The thermodynamic balance between these “Up” and “Down” populations would be a major determinant of the fluorescence performances, due to several possible mechanisms outlined below.

First of all, the “Down” configuration ensures permanent H-bonding of residue 65 to a well conserved water molecule, whose role might be important in controling chromophore flexibility. A water molecule lying similarly below the imidazolinone ring is found in most crystallographic structures of fluorescent proteins from *Aequorea* and other natural sources [59]–[61]. In *Aequorea* variants, this water molecule is the first of a coplanar layer of 3 to 5 H-bonded water molecules lining parallel to the chromophore (Figure 8), that is likely one of the first obstacles against chromophore torsion on this side. Indeed, during molecular dynamic simulations of GFP-S65T, we found that this water layer was disrupted upon excited-state torsion (unpublished data). Strikingly, this water layer is highly conserved (and quite similar to that of *Av*GFP) in all CFP structures adopting the “Down” configuration, while it shows a higher degree of variability for CFP variants in the “Up” configuration (Figure S11).

The “Up” configuration not only disconnects residue 65 from this important water layer, but also allows the formation of new H-bonds to two alternate, mutually exclusive proton acceptors: the imidazolinone nitrogen and the valine 61 carbonyl oxygen. Direct intra-chromophore H-bonding between residue 65 and the imidazolinone nitrogen may be particularly perturbing to the chromophore structure and electronic conjugaison. Alternatively, H-bonding to valine 61 may result in some new strain on the local main chain configuration. One or both of these interactions may thus have a quite significant influence on chromophore flexibility and photophysics.

Finally, the H-bonding status of the residue 65 hydroxyl while in the “Up” configuration is likely to be under tight control of the protonation state of Glu222. In all GFP and CFP structures, and regardless of the Up/Down configurations, at least one of the two oxygen atoms of Glu222 is found in H-bonding distance of the residue 65 hydroxyl (Figure S11). In its deprotonated, charged state, Glu222 might become the favorite proton acceptor of this hydroxyl. In this case, the residue 65 in the “Up” configuration will not be able to participate in the destabilizing interactions with the imidazolinone nitrogen and/or valine 61 carbonyl. These unfavorable interactions would be more accessible at acid pHs, when Glu222 becomes permanently protonated, which would account for the strong pH sensitivity of CFP variants with a threonine in position 65.

To summarize, the crystallographic data on ECFP and Cerulean provides indications for a conformational switch of residue 65 between well defined “Up” and “Down” configurations, the “Down” configuration being more rigid and more similar to the native configuration of *Av*GFP, and the “Up” configuration associated to multiple destabilization factors. It may be speculated that these two configurations bear some relationship with the long fluorescence lifetime on one hand, and the variable set of short lifetime components on the other, that are separated in the fluorescence decays of all CFP variants. Shifts in the thermodynamic equilibrium between these two conformations in solution would therefore comprehensively account for all photophysical consequences of the T65S mutation, including decreases in the fluorescence emission heterogeneity (as revealed by time-resolved fluorescence spectroscopy) and in the sensitivity to pH.

### Conclusion

The development of greatly improved CFP variants is an important breakthrough for accurate and sensitive FRET analyses of biochemistry inside the living cell [6]. It is particularly important for FRET-FLIM applications, where the quantification of FRET heavily relies upon the CFP donor fluorescence properties. Besides its improved brightness, we show here that the recently developed variant mTurquoise [13] has an almost pure single exponential fluorescence decay, an outstanding stability in the acid pH range and a greatly improved photostability both in terms of reversible and irreversible responses. Our results also show that it is possible to strongly enhance the performances of numerous existing genetic constructs based on ECFP or Cerulean, by introducing the T65S single point mutation. In the case of Cerulean, this will alleviate most of its remaining drawbacks, and will result in performances nearly equivalent to those of mTurquoise. We recently expressed successfully a variety of CFP constructs carrying the T65S mutation in different mammalian cells, and obtained substantial improvements in intracellular brightness, photostability and environmental insensitivity of the corresponding reporters (Erard et al, submitted).

Our study establishes a strong relationship between the local protein flexibility, excited state chromophore isomerization, and the general fluorescence performances, including brightness, environmental sensitivity and photostability. Moreover, it pinpoints the possible crucial role of residue 65 and its variable H-bonding status in controling this photophysics, a finding that now requires further structural and dynamic studies. It will be interesting also to evaluate the interplay between this dynamics and the other mutations carried by improved CFP variants. Although these results only pertain to cyan fluorescent proteins, it is striking to note that all structural elements presumably involved in the effects of the T65S mutation are present in other members of the *Av*GFP family. Therefore, our findings may have a more general relevance, that is open for future works. This is particularly interesting, as the near ultimate photophysical performances now reached by CFPs would suggest a route towards the similar engineering of other popular fluorescent proteins.

## Supporting Information

Figure S1 **Absorption spectra of CFP variants at basic, neutral and acid pHs.** (A–E) Absorption spectra of the different CFP variants. (F) Model spectra obtained by linear combinations of the native and denatured spectra, showing the range of possible spectral shapes in the hypothesis of a two-state transition (see Text S1). The percentages indicate the relative contribution of the denatured form to the spectrum, that may differ from its relative population. These spectra should not be used to locate possible isosbestic points.
(TIF)

Figure S2 **Fluorescence emission spectra of CFP variants at basic, neutral and acid pHs.** (A–E) Fluorescence emission spectra of the different CFP variants. (F) Model spectra obtained by linear combinations of the native and denatured spectra, showing the range of possible spectral shapes in the hypothesis of a two-state transition. The percentages indicate the relative contribution of the denatured form to the spectrum, which differs substantially from its relative population (see Text S1).
(TIF)

Figure S3 **Secondary structure content of CFP variants at neutral and acid pHs.** Acid pHs mainly result in a loss of 10% of β-sheet, the main component of the native protein structure at pH 7.4.
(TIF)

Figure S4 **Photoactivated return of ECFP fluorescence after transient photobleaching.** The ECFP fluorescence was first bleached by maximum lamp power for less than 1 min. The return of fluorescence after switching off the illumination, was then monitored under different illumination regimes, and the different transient responses were normalized between minimum and maximum fluorescence levels: normalized experimental data (markers) and best fits (continuous lines) to the model F^norm^ =  y_0_+y_1_t+y_2_ exp(−t/τ_Back_) (see Text S1).
(TIF)

Figure S5 **Dependence of the reversible bleaching rate constants of ECFP on the irradiance.**
(TIF)

Figure S6 **Dependence of the amplitude of reversible bleaching of ECFP on the irradiance.**
(TIF)

Figure S7 **Apparent photobleaching and recovery of ECFP fluorescence under variable illumination conditions.** Experiments designed to reproduce the results of [14]. See Text S1.
(TIF)

Figure S8 **Reversible photobleaching of cytosolic CFPs expressed in living MDCK cells.** Experimental conditions were identical to those used for purified proteins. Each curve is an average of 4 to 6 decays collected from different cell individuals. Continuous lines are best fits to the model F^norm^ =  y_0_+y_1_t+y_2_ exp(−t/τ_Rev_).
(TIF)

Figure S9 **Irreversible bleaching of cytosolic CFPs expressed in living MDCK cells.** (A) Constant irradiation at 0.2 W/cm^2^ was applied while camera images were taken every 20 s. Each curve is the average of 4 to 6 decays collected from different cell individuals. Continuous lines are best fits of the decays to a simple exponential model with time constant τ_Irrev_. (B) Dependence of the irreversible bleaching rates of ECFP on the irradiance.
(TIF)

Figure S10 **Stereogram of the overlapped structures of mTurquoise and SCFP3A in the CFP chromophore region.** The structures of mTurquoise (2YE0, solid), and SCFP3A (2YDZ, transparent) [15] were aligned along the protein backbones. All heavy atoms and water molecules located within 9 Å of the CG atom of the chromophore are displayed. RMSD calculations were performed after further alignment of this set of atoms.
(TIF)

Figure S11 **H-bonding networks in the chromophore cavity of ECFP, Cerulean and GFP.** (A) ECFP structure of Lelimousin et al (2WSN, solid, grey) [40] and Bae *et* *al* (1OXD, transparent, red) [39], reproduced from the Main Section, (B) Cerulean structure of Lelimousin *et* *al* (2WSO, solid, grey) and Malo et al (2Q57, transparent, red) [29], the latter displaying an anusual “*trans*” isomer of the chromophore, and (C) *Av*GFP structure (1W7S, solid, grey) [62], and GFP-S65T structure (1Q4A, transparent, red) [57]. Overlayed structures were aligned along the whole protein backbones, water oxygens are shown as spheres and major H-bonds as dashed lines.
(TIF)

Table S1 **Complementary time-resolved fluorescence parameters of CFP variants.** Correlation of the CFP fluorescence quantum yields with the integrated pre-exponential amplitude (a_L_) and position (τ_L_) of the longest lifetime peak in fluorescence lifetime distributions.
(DOC)

Table S2 **Reversible bleaching parameters of purified and cytosolic CFP variants.** The elementary rate constants of reversible photobleaching k_off_ and photoactivated return k_on_ were determined from experimental data on agarose beads as described in Text S1.
(DOC)

Text S1 I. Spectral analyses of CFP variants at different pHs. II. Modeling and analysis of photobleaching experiments. III. Structural analyses of the chromophore environment.
(DOC)
