# Supplementary figures and images for: The Single T65S Mutation Generates Brighter Cyan Fluorescent Proteins with Increased Photostability and pH Insensitivity
Source: PLoS One. 2012 Nov 2;7(11):e49149. doi: 10.1371/journal.pone.0049149 (PMC3487735; doi:10.1371/journal.pone.0049149)

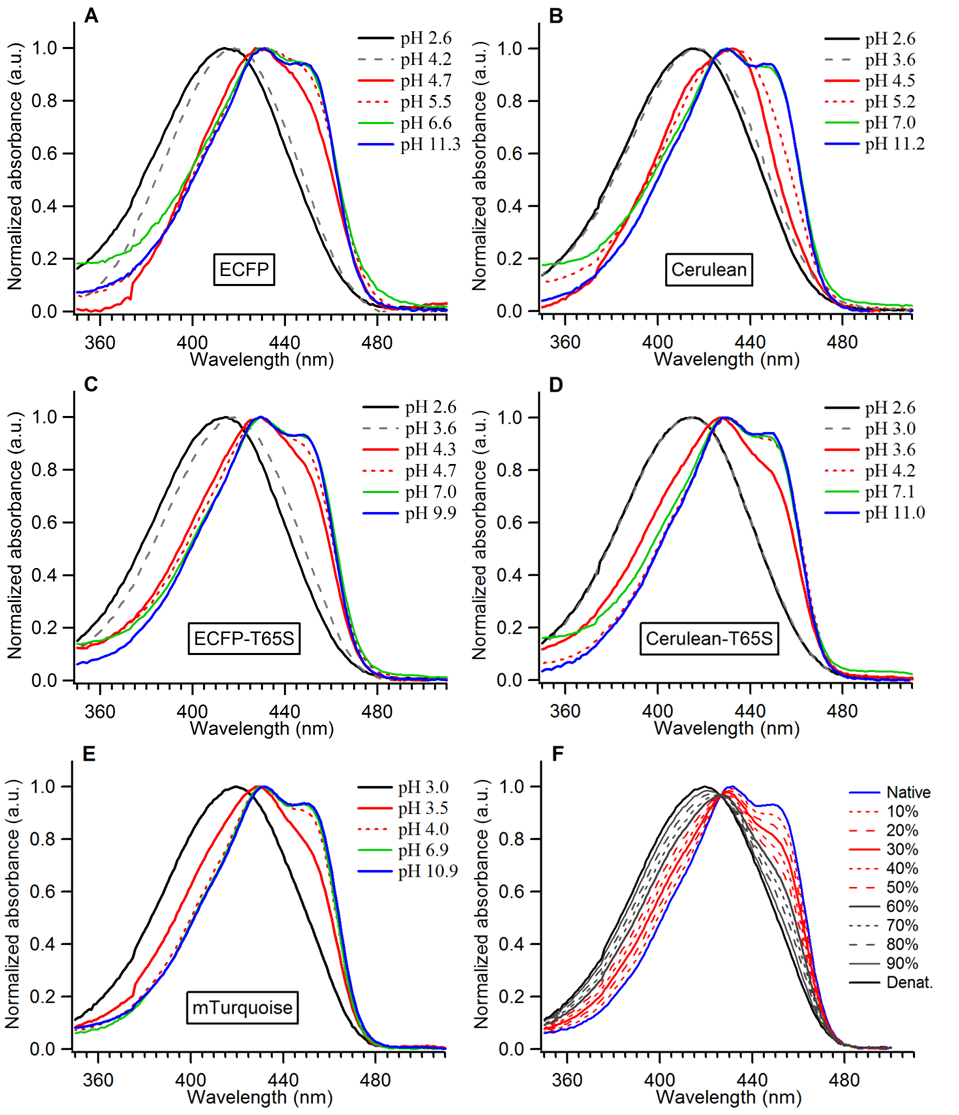

Supplement: Figure S1 — Absorption spectra of CFP variants at basic, neutral and acid pHs. (A–E) Absorption spectra of the different CFP variants. (F) Model spectra obtained by linear combinations of the native and denatured spectra, showing the range of possible spectral shapes in the hypothesis of a two-state transition (see Text S1). The percentages indicate the relative contribution of the denatured form to the spectrum, that may differ from its relative population. These spectra should not be used to locate possible isosbestic points. (TIF) [file pone.0049149.s001.tif]

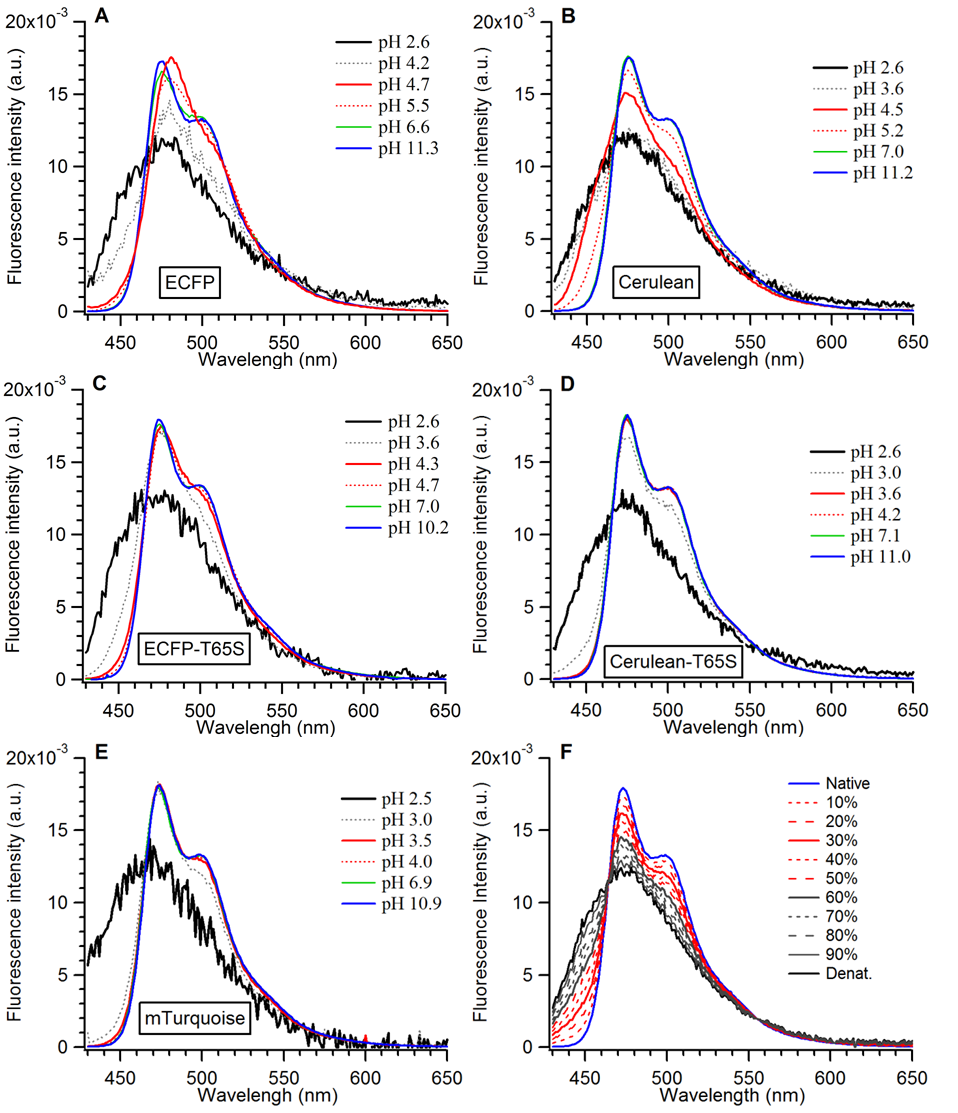

Supplement: Figure S2 — Fluorescence emission spectra of CFP variants at basic, neutral and acid pHs. (A–E) Fluorescence emission spectra of the different CFP variants. (F) Model spectra obtained by linear combinations of the native and denatured spectra, showing the range of possible spectral shapes in the hypothesis of a two-state transition. The percentages indicate the relative contribution of the denatured form to the spectrum, which differs substantially from its relative population (see Text S1). (TIF) [file pone.0049149.s002.tif]

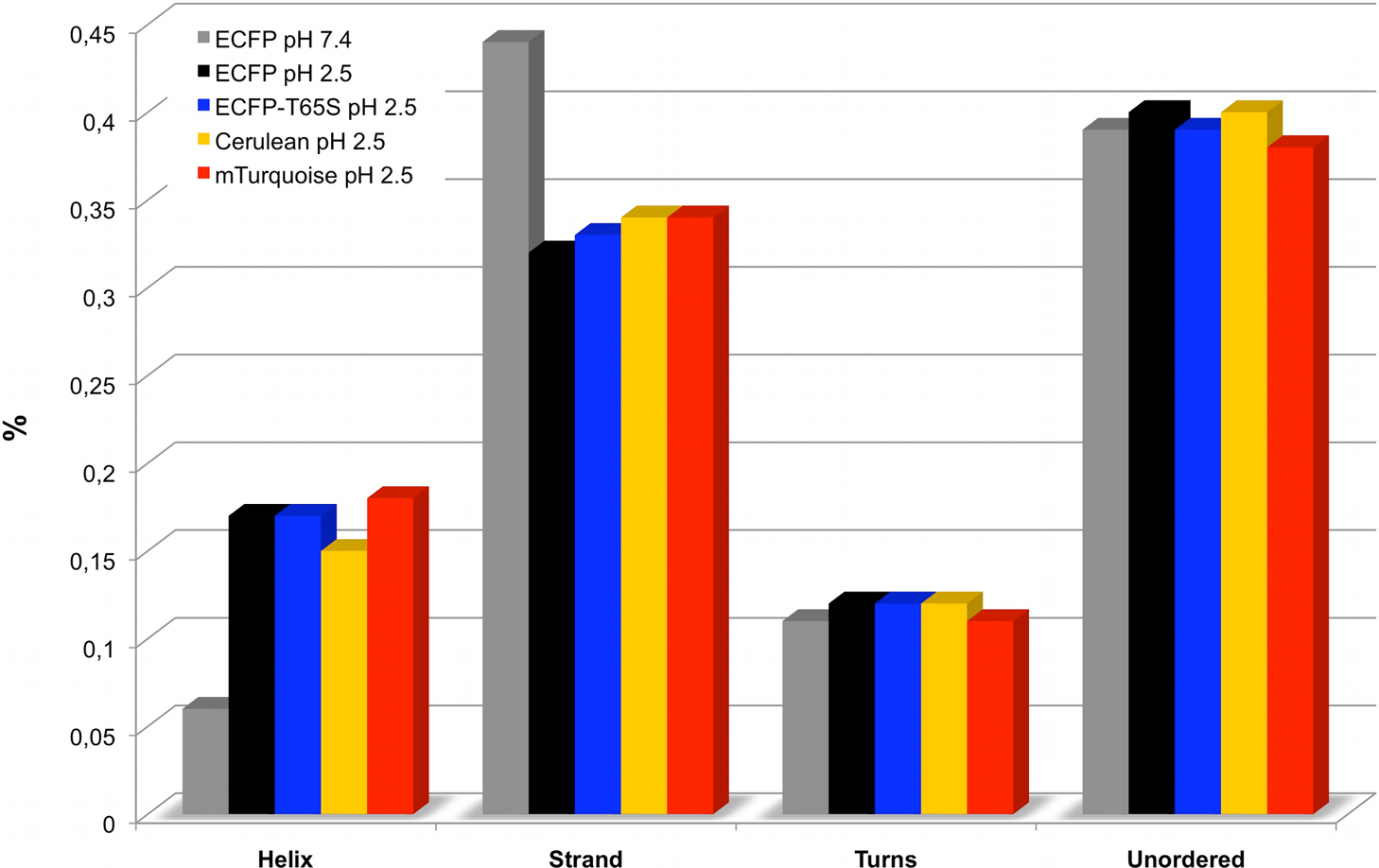

Supplement: Figure S3 — Secondary structure content of CFP variants at neutral and acid pHs. Acid pHs mainly result in a loss of 10% of β-sheet, the main component of the native protein structure at pH 7.4. (TIF) [file pone.0049149.s003.tif]

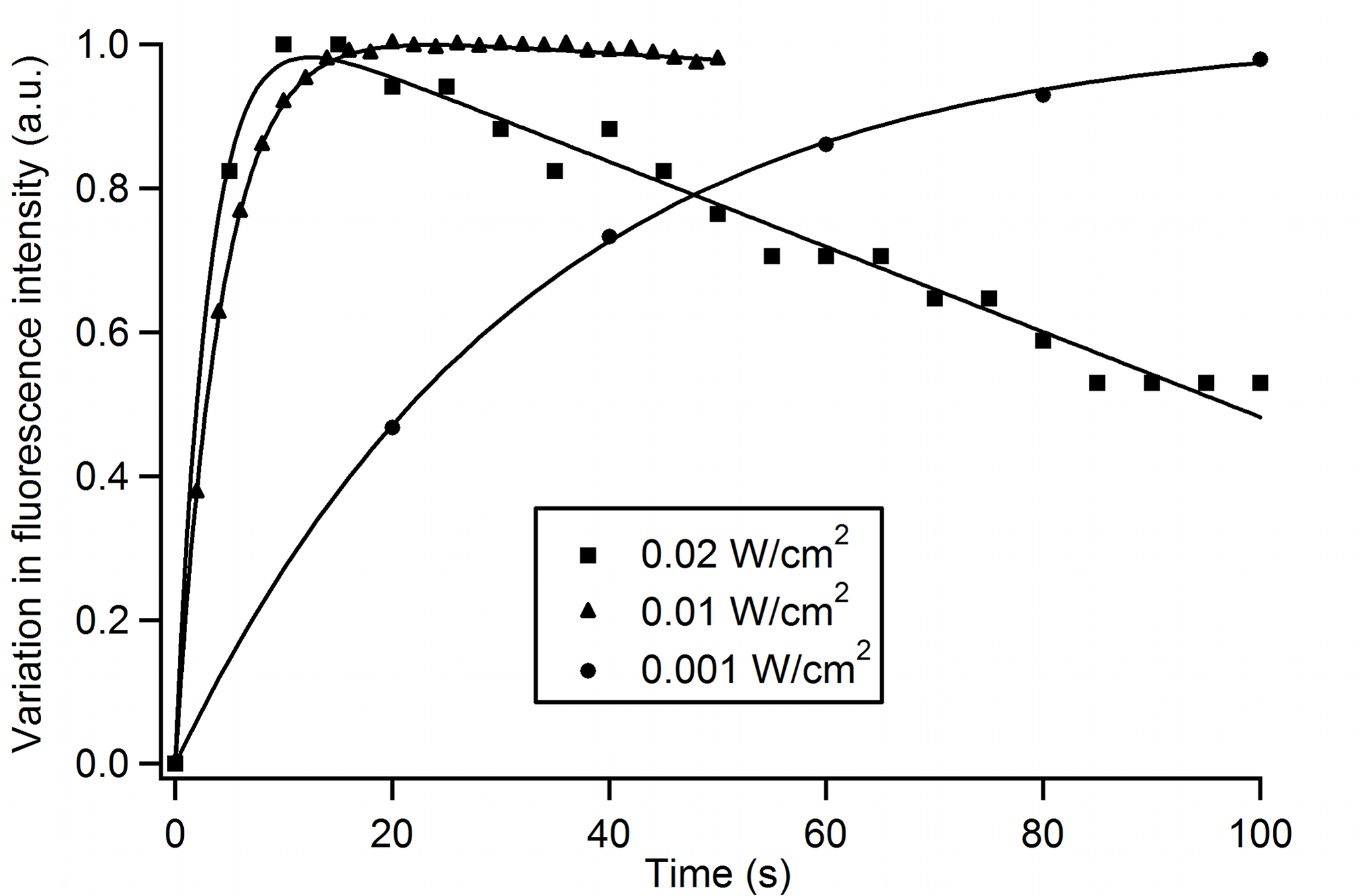

Supplement: Figure S4 — Photoactivated return of ECFP fluorescence after transient photobleaching. The ECFP fluorescence was first bleached by maximum lamp power for less than 1 min. The return of fluorescence after switching off the illumination, was then monitored under different illumination regimes, and the different transient responses were normalized between minimum and maximum fluorescence levels: normalized experimental data (markers) and best fits (continuous lines) to the model Fnorm = y0+y1t+y2 exp(−t/τBack) (see Text S1). (TIF) [file pone.0049149.s004.tif]

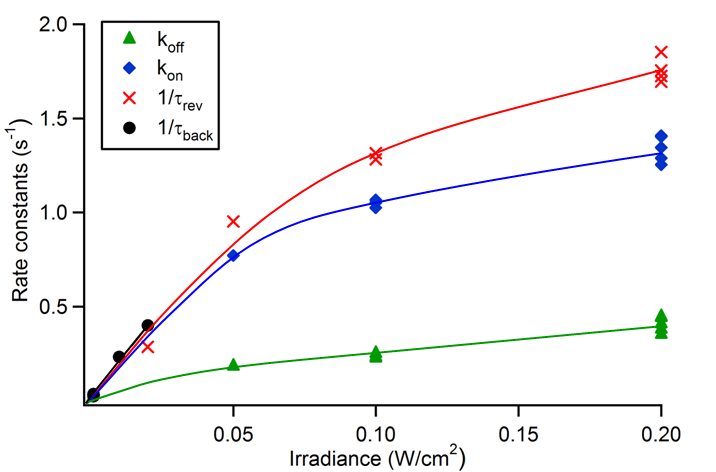

Supplement: Figure S5 — Dependence of the reversible bleaching rate constants of ECFP on the irradiance. (TIF) [file pone.0049149.s005.tif]

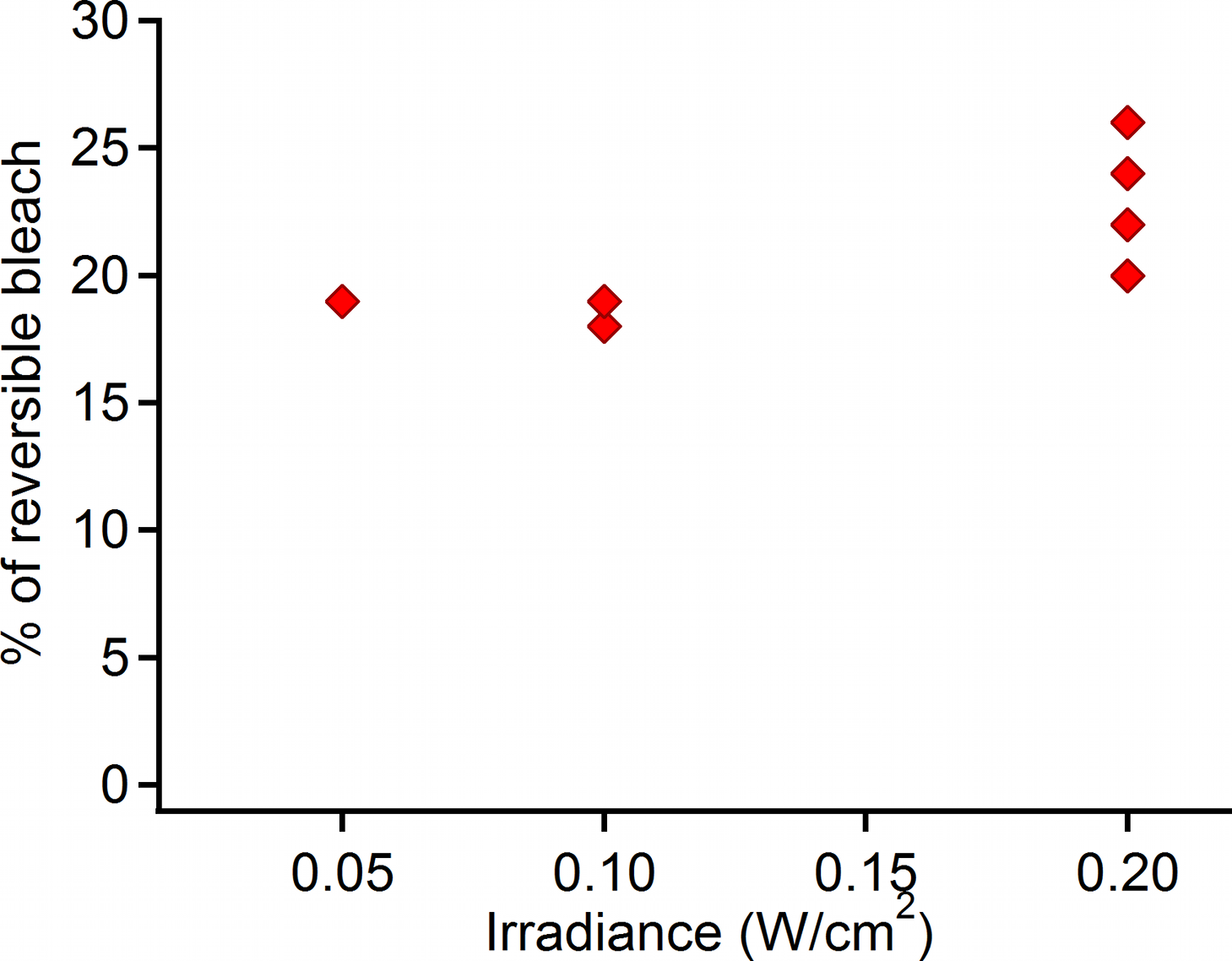

Supplement: Figure S6 — Dependence of the amplitude of reversible bleaching of ECFP on the irradiance. (TIF) [file pone.0049149.s006.tif]

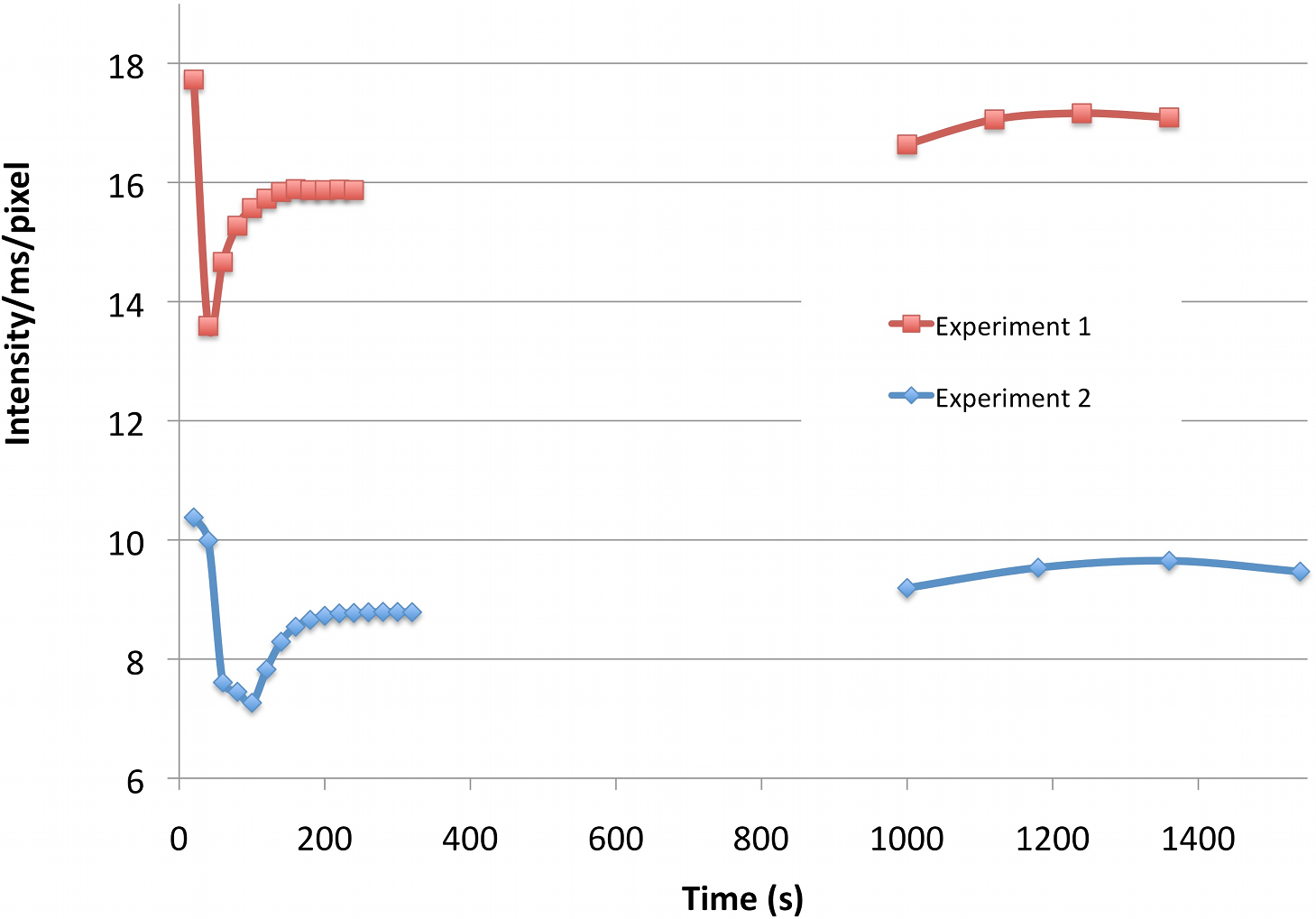

Supplement: Figure S7 — Apparent photobleaching and recovery of ECFP fluorescence under variable illumination conditions. Experiments designed to reproduce the results of [14]. See Text S1. (TIF) [file pone.0049149.s007.tif]

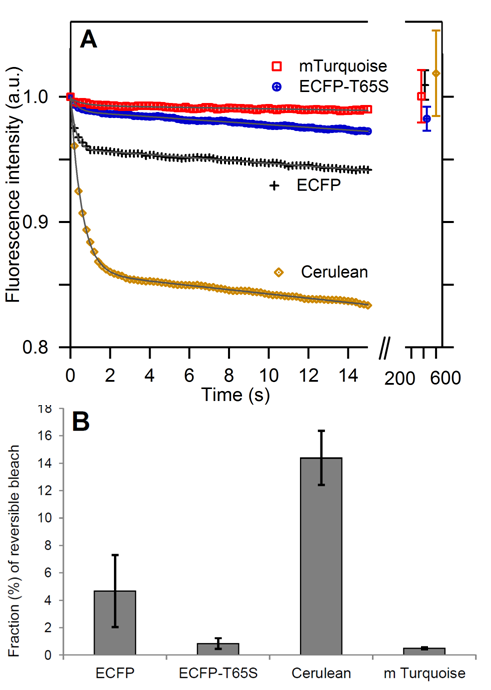

Supplement: Figure S8 — Reversible photobleaching of cytosolic CFPs expressed in living MDCK cells. Experimental conditions were identical to those used for purified proteins. Each curve is an average of 4 to 6 decays collected from different cell individuals. Continuous lines are best fits to the model Fnorm = y0+y1t+y2 exp(−t/τRev). (TIF) [file pone.0049149.s008.tif]

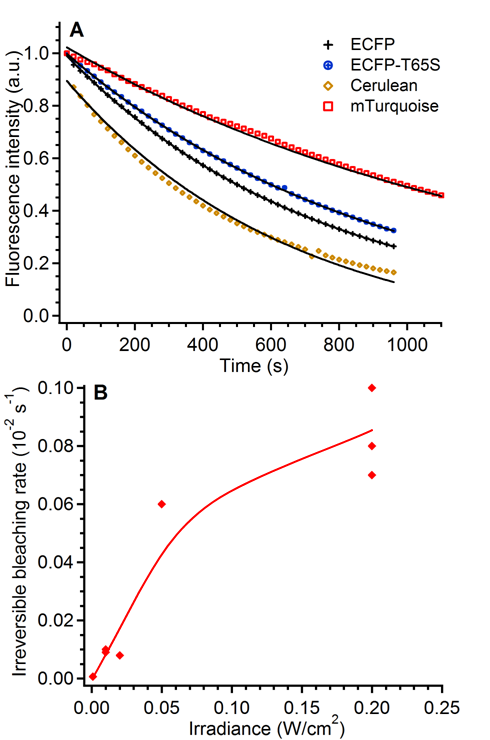

Supplement: Figure S9 — Irreversible bleaching of cytosolic CFPs expressed in living MDCK cells. (A) Constant irradiation at 0.2 W/cm2 was applied while camera images were taken every 20 s. Each curve is the average of 4 to 6 decays collected from different cell individuals. Continuous lines are best fits of the decays to a simple exponential model with time constant τIrrev. (B) Dependence of the irreversible bleaching rates of ECFP on the irradiance. (TIF) [file pone.0049149.s009.tif]

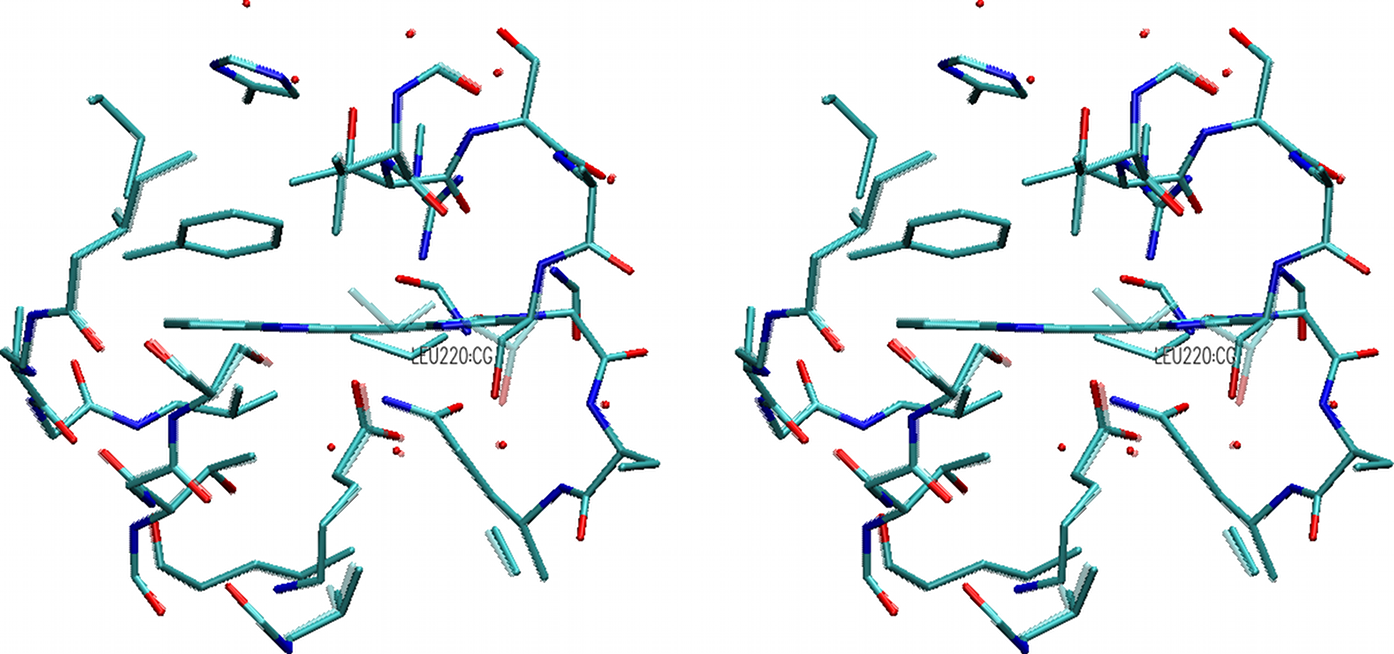

Supplement: Figure S10 — Stereogram of the overlapped structures of mTurquoise and SCFP3A in the CFP chromophore region. The structures of mTurquoise (2YE0, solid), and SCFP3A (2YDZ, transparent) [15] were aligned along the protein backbones. All heavy atoms and water molecules located within 9 Å of the CG atom of the chromophore are displayed. RMSD calculations were performed after further alignment of this set of atoms. (TIF) [file pone.0049149.s010.tif]

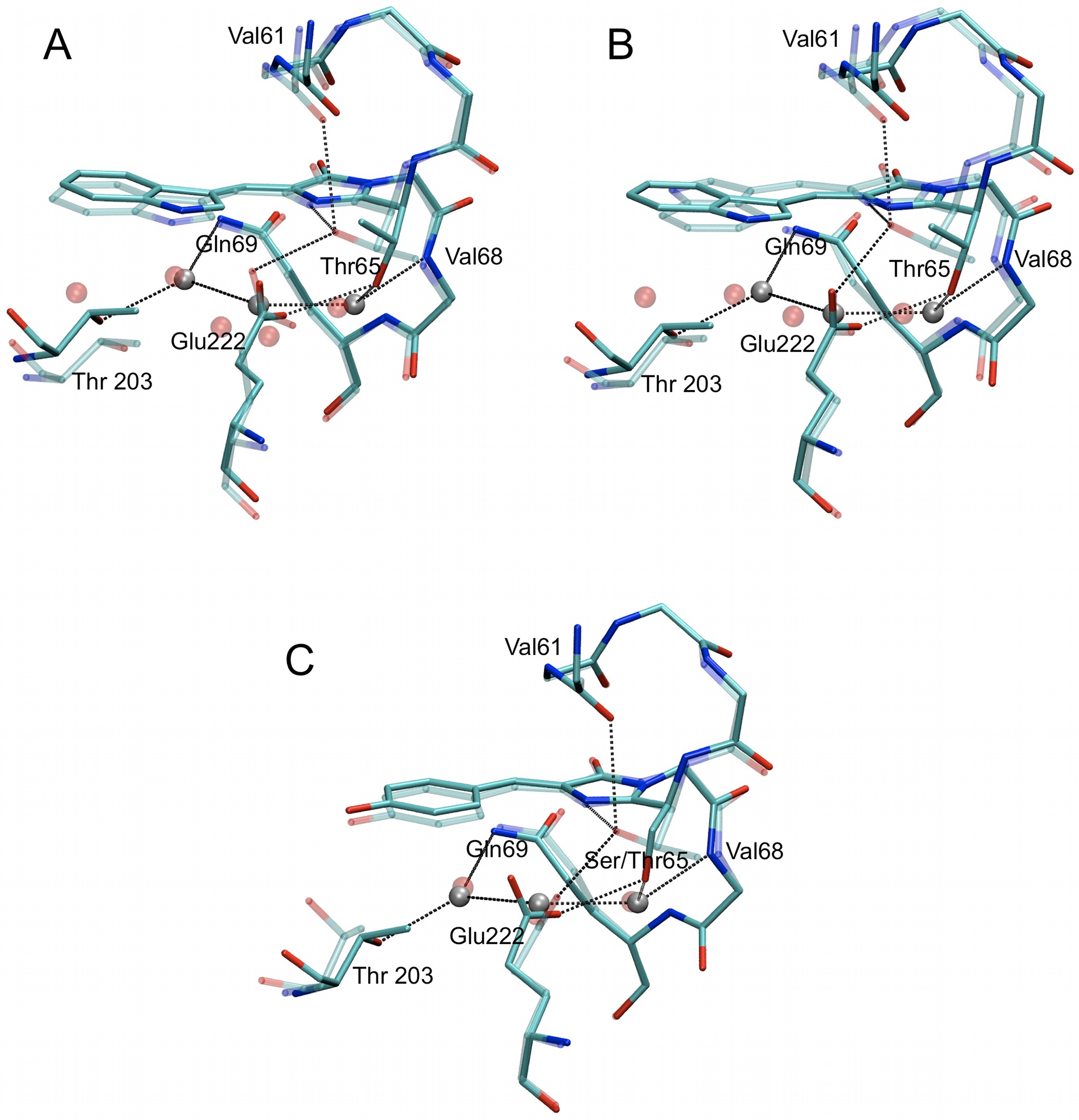

Supplement: Figure S11 — H-bonding networks in the chromophore cavity of ECFP, Cerulean and GFP. (A) ECFP structure of Lelimousin et al (2WSN, solid, grey) [40] and Bae et al (1OXD, transparent, red) [39], reproduced from the Main Section, (B) Cerulean structure of Lelimousin et al (2WSO, solid, grey) and Malo et al (2Q57, transparent, red) [29], the latter displaying an anusual “trans” isomer of the chromophore, and (C) AvGFP structure (1W7S, solid, grey) [62], and GFP-S65T structure (1Q4A, transparent, red) [57]. Overlayed structures were aligned along the whole protein backbones, water oxygens are shown as spheres and major H-bonds as dashed lines. (TIF) [file pone.0049149.s011.tif]
