# Supplementary material for: The Single T65S Mutation Generates Brighter Cyan Fluorescent Proteins with Increased Photostability and pH Insensitivity
Source: PLoS One. 2012 Nov 2;7(11):e49149. doi: 10.1371/journal.pone.0049149 (PMC3487735; doi:10.1371/journal.pone.0049149)
Supplement: Table S1 — Complementary time-resolved fluorescence parameters of CFP variants. Correlation of the CFP fluorescence quantum yields with the integrated pre-exponential amplitude (aL) and position (τL) of the longest lifetime peak in fluorescence lifetime distributions. (DOC) [file pone.0049149.s012.doc]

**Table S1. Complementary time-resolved fluorescence parameters of CFP variants**

| Protein | Quantum Yield | τ_L_ (ns)  ± Std Dev | a_L_ (%)  ± Std Dev |
| --- | --- | --- | --- |
| ECFP | 0.40 | 3.51 ± 0.12 | 52 ± 7 |
| ECFP-T65S | 0.59 | 3.78 ± 0.11 | 77 ± 8 |
| Cerulean | 0.67 | 3.80 ± 0.13 | 64 ± 7 |
| Cerulean-T65S | 0.84 | 4.17 ± 0.09 | 83 ± 11 |
| mTurquoise | 0.85 | 4.26 ± 0.08 | 87 ± 10 |

Correlation of the CFP fluorescence quantum yields with the integrated pre-exponential amplitude (a_L_) and position (τ_L_) of the longest lifetime peak in fluorescence lifetime distributions.
