# Supplementary material for: The Single T65S Mutation Generates Brighter Cyan Fluorescent Proteins with Increased Photostability and pH Insensitivity
Source: PLoS One. 2012 Nov 2;7(11):e49149. doi: 10.1371/journal.pone.0049149 (PMC3487735; doi:10.1371/journal.pone.0049149)
Supplement: Table S2 — Reversible bleaching parameters of purified and cytosolic CFP variants. The elementary rate constants of reversible photobleaching koff and photoactivated return kon were determined from experimental data on agarose beads as described in Text S1. (DOC) [file pone.0049149.s013.doc]

**Table S2. Reversible bleaching parameters of purified and cytosolic CFP variants.**

| Protein | Agarose beads | | | | Living cells | | |
| --- | --- | --- | --- | --- | --- | --- | --- |
|  | % Rev  ± 2% | τ_Rev_  (s) ± 0.1 | k_off_  (s^-1^) | k_on_  (s^-1^) | % Rev  ± Std Dev | τ_Rev_  ± Std Dev (s) | N_cell_ |
| ECFP | 23.1 | 0.6 | 0.404 | 1.35 | 5±3 | 0.28±0.04 | 21 |
| ECFP-T65S | 3.0 | 1.0 | 0.029 | 0.94 | 0.8±0.4 | 0.6±0.3 | 15 |
| Cerulean | 33.0 | 1.0 | 0.337 | 0.68 | 14±2 | 0.6±0.1 | 21 |
| Cerulean-T65S | 2.5 | 0.8 | 0.033 | 1.30 | ND | ND |  |
| mTurquoise | 0.6 | 1.4 | 0.004 | 0.71 | 0.6±0.20 | 0.6±0.3 | 14 |

Amplitudes and rate constants of reversible bleaching determined as described in Supplementary Text S1.
