# Supplementary material for: The Single T65S Mutation Generates Brighter Cyan Fluorescent Proteins with Increased Photostability and pH Insensitivity
Source: PLoS One. 2012 Nov 2;7(11):e49149. doi: 10.1371/journal.pone.0049149 (PMC3487735; doi:10.1371/journal.pone.0049149)
Supplement: Text S1 — I. Spectral analyses of CFP variants at different pHs. II. Modeling and analysis of photobleaching experiments. III. Structural analyses of the chromophore environment. (DOC) [file pone.0049149.s014.doc]

SUPPLEMENTARY TEXT INFORMATION

**Table S1. Complementary time-resolved fluorescence parameters of CFP variants**

| Protein | Quantum Yield | τ_L_ (ns)  ± Std Dev | a_L_ (%)  ± Std Dev |
| --- | --- | --- | --- |
| ECFP | 0.40 | 3.51 ± 0.12 | 52 ± 7 |
| ECFP-T65S | 0.59 | 3.78 ± 0.11 | 77 ± 8 |
| Cerulean | 0.67 | 3.80 ± 0.13 | 64 ± 7 |
| Cerulean-T65S | 0.84 | 4.17 ± 0.09 | 83 ± 11 |
| mTurquoise | 0.85 | 4.26 ± 0.08 | 87 ± 10 |

Correlation of the CFP fluorescence quantum yields with the integrated pre-exponential amplitude (a_L_) and position (τ_L_) of the longest lifetime peak in fluorescence lifetime distributions.

1. S*pectral analyzes of CFP variants at different pHs*

*Absorption spectra*

The absorption spectra of the different CFP variants at basic, neutral and acids pHs are shown in Figure S1(A-E). Spectra are normalized at the maximum absorbance peak. The intermediate spectrum most distant from neutral and acid spectra is shown by a continuous red line. Depending on the variant, its pH may or not coincide with the pK_1/2_ of fluorescence intensity loss. The model spectra of Figure S1F assume increasing contributions of the acid *vs* neutral absorption spectra of mTurquoise. They show that, in the case of a simple two-state transition, a significant blue expansion of the absorption spectrum must take place *simultaneously* to the loss of two-hump structure on the red side, and before any major blue shift of the peak absorbance.

In the case of ECFP (Figure S1A), a near-complete loss of structure on the red side is observed at pH 4.7, before the spectrum shifts or expands to the blue. For the other CFP variants (Figures S1B-E), the loss of two-hump structure coincides with some blue expansion. In the case of mTurquoise and Cerulean-T65S, the absorption spectrum obtained at pH=pK_1/2_ is very similar to a 50/50 mixture of typical neutral and acid spectra.

*Fluorescence spectra*

The fluorescence emission spectra of the different CFP variants at basic, neutral and acids pHs are shown in Figure S2(A-E). Spectra are normalized to unit surface. The intermediate spectrum most distant from neutral and acid spectra is shown with a continuous red line. Depending on the variant, its pH may or not coincide with the pK_1/2_ of fluorescence intensity loss. The model spectra of Figure S2F assume increasing contributions of the acid *vs* neutral fluorescence spectra of mTurquoise. However, because of the very different fluorescence quantum yields of the acid and neutral forms, a fluorescence spectrum characteristic of 50/50 contributions is expected well below the pK_1/2_ of mTurquoise.

In the case of ECFP (Figure S2A), below pH 6 and down to pH 4.7, the fluorescence maximum transiently shifts to the red by 10nm, while the spectrum looses its two-hump structure. At pH 4.2, the spectrum broadens, with now a significant contribution on the blue edge, likely characteristic of the final denatured state. This intermediate red shift of the emission maximum is unique to the ECFP form. Instead, and in the case of Cerulean only (Figure S2C), a marked shoulder appears on the blue edge at pHs close to 4, an already reported signature of chromophore isomerization [1]. Conversely, both Cerulean-T65S and mTurquoise still display a structured emission spectrum at pH 3 (Figure S2D,E), a pH at which they have lost more than 90% of their fluorescence intensity (Figure 3, Main Section), which points to the outstanding acid stability of these two variants.

1. *Modeling and analyzis of photobleaching experiments*

**Table S2. Reversible bleaching parameters of purified and cytosolic CFP variants.**

| Protein | Agarose beads | | | | Living cells | | |
| --- | --- | --- | --- | --- | --- | --- | --- |
|  | % Rev  ± 2% | τ_Rev_  (s) ± 0.1 | k_off_  (s^-1^) | k_on_  (s^-1^) | % Rev  ± Std Dev | τ_Rev_  ± Std Dev (s) | N_cell_ |
| ECFP | 23.1 | 0.6 | 0.404 | 1.35 | 5±3 | 0.28±0.04 | 21 |
| ECFP-T65S | 3.0 | 1.0 | 0.029 | 0.94 | 0.8±0.4 | 0.6±0.3 | 15 |
| Cerulean | 33.0 | 1.0 | 0.337 | 0.68 | 14±2 | 0.6±0.1 | 21 |
| Cerulean-T65S | 2.5 | 0.8 | 0.033 | 1.30 | ND | ND |  |
| mTurquoise | 0.6 | 1.4 | 0.004 | 0.71 | 0.6±0.20 | 0.6±0.3 | 14 |

Amplitudes and rate constants of reversible bleaching determined as described below.

*Kinetic model of a simple two-state reaction*

We assume that CFP undergoes a reversible photoactivated reaction between two states, one fluorescent and the other not, characterized by the reaction rates k_off_ and k_on_.


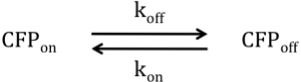


The model neglects thermal relaxation between these states, as well as irreversible photobleaching (both taking place on slower time scales, under our experimental conditions), and assumes identical absorption of both forms. It leads to the following rate equations :

(Eq. S1)

and taking x_0_=1 (no off state at time zero, when the system is at thermal equilibrium in the dark), one gets :

(Eq. S2)

i.e., upon sudden illumination, the molar fraction of the fluorescent state decreases following a single exponential with time constant τ_Rev_ = 1/(k_on_+k_off_), towards a steady state level y_0_ given by k_on_/(k_on_+k_off_).

*Fitting of reversible bleaching experiments*

After normalization to unit initial intensity, the transient bleaching curves were fitted to the model :

where y_0_ stands for the steady-state fluorescence level, y_1_ for a linear irreversible photobleaching rate (fitted values of y_1_ were usually ≈-1*10^-3^ s^-1^, in agreement with our experimental irreversible photobleaching rates, see Table 1, Main Section), while y_2_ and τ_Rev_ are respectively the relative amplitude and time constant of the reversible bleaching.

*Fluorescence recovery after transient photobleaching is accelerated under illumination*

After normalization from zero initial intensity to unit maximum intensity, the fluorescence recovery curves were fitted to the same analytical model :

The relaxation times for fluorescence recovery τ_Back_ are in excellent agreement with relaxation times for loss of fluorescence τ_Rev_ (see Figure S5).

*All rate constants depend on the illumination intensity*

The rate constants k_on_ and k_off_ are directly obtained from y_0_ and τ_Rev_ :

(Eq. S3)

According to the two-state model, assuming a linear dependence of k_on_ and k_off_ on the illumination power, the relaxation time τ_Rev_ should also linearly depend on the excitation power (or irradiance, in W/cm^2^), while the fraction of reversible fluorescence loss (1- y_0_ ) should not, which is approximately verified for low excitation power (Figures S5 and S6).

*Quantum efficiences of ECFP photoconversion*

The quantum efficiencies φ_off_ and φ_on_ of the CFP photoconversion reactions are given by :

(Eq. S4)

where k_exc_ is the excitation rate per molecule, given by the product of the absorption cross-section σ_exc_ by the photon flux N_exc_ at the excitation wavelength λ_exc_:

k_exc_= σ_exc_(cm^2^) x N_exc_ (Photons/s/cm^2^)

For ECFP, with I_exc_ = 0.05 W/cm^2^, assuming ε_on_= ε_off_≈30000 M^-1^cm^-1^ at λ_exc_=440 nm, we obtain k_exc_= 13 s^-1^, with k_off_ = 0.18 s^-1^ and k_on_ = 0.77 s^-1^, giving φ_off_ = 1.4% and φ_on_ =6.1%. These values are significantly higher than those reported by Sinnecker and colleagues [2], which we chiefly ascribe to differences in illumination intensity. Indeed, the irradiance used by Sinnecker et al for quantum efficiency determination (reportedly : 3.5 W/cm^2^ at 460 nm, see their Figure 4) lies at least 5 times above the onset of saturation in their case (see their Figure 5).

*Comparison with the experiments of Markwardt et al [3]*

Using variable irradiation sequences, we were able to qualitatively reproduce on ECFP reversible bleaching experiments very similar to those of Rizzo and coworkers [3] in their Figure 3 (Figure S7, left side). Agarose beads labeled with ECFP were first submitted to irradiation at 0.2 W/cm2 for 10 s (Experiment 1) or 1 min (Experiment 2). The recovery of fluorescence was then monitored by taking camera images while the illumination time was restricted to camera exposure times (100 ms every 20 s), corresponding to an average irradiance of 10^-3^ W/cm^2^. To complete these experiments, after 10 min in the dark, a few camera images were taken again, to check the final fluorescence level (Figure S7, right side).

The additional fluorescence recovery after a long dark period shows that this type of experiment underestimates the proportion of reversible bleaching, and gives erroneous indications on the irreversible bleaching component. While the amount of "irreversible" bleaching is not in proportion of the irradiation dose (compare Experiment 1 and Experiment 2), the fluorescence recovery phase, because of illumination of the sample while taking camera images, corresponds to a mixture of photoactivated "off" and "on" reactions and of thermal recovery (not negligible in this case).

1. *Structural analyzes of the chromophore environment*

In wild-type *Av*GFP, the serine 65 hydroxyl is connected to the phenol hydroxyl of the chromophore via an important continuous wire of hydrogen bonds which has been extensively discussed in the literature (for a review see [4]). In CFPs, where the chromophore phenol is replaced by an indole moiety, this proton wire is absent, but another chain of tight hydrogen bonds, connecting the indole nitrogen to the threonine 65 hydroxyl via Ser205 and Glu222, strongly hampers the chromophore torsions. However, to the exception of Cerulean at acid pH (2Q57), where the chromophore indole is rotated away due to an unusual *trans* configuration [1], we find that this chain of H-bonds is well preserved in all CFP structures. On the other hand, when going from SCFP3A to mTurquoise, a slight increase in the overall stability of the chromophore pocket, by about 1.5 kcal/mol, has been reported and ascribed to the T65S mutation [5]. However, this increased stability can hardly be traced to any significant structural difference in the chromophore surroundings of the two proteins (Figure S10), and thus a causal relationship with the major changes in fluorescence properties associated with the T65S mutation is not obvious. Such a stabilization might nevertheless favor a greater proportion of the crystallographic configuration of mTurquoise in solution, as compared to SCFP3A, a fact that would be fully consistent with our Up/Down thermodynamic balance hypothesis.

We then analyzed in more detail the direct interactions of residue 65 in the two currently available structures of ECFP (1OXD, 2WSN), and Cerulean (2Q57, 2WSO). This was completed by similar analyzes of the structures of *Av*GFP variants carrying either a serine in position 65 (1GFL, 1EMB, 1W7S, 2WUR, 2DUI, 3GJ1, 1HCJ) or a threonine (1EMA, 1EMG, 1C4F, 1Q4A, 1Q4B, 2Y0G). Structures were aligned first along the whole protein backbones, and then the details of the chromophore pocket were examined. More than their differences, the most striking features of all these structures is their remarkably close similarity : in all cases, and despite large differences in the chromophore structure (between CFPs and GFPs) or conformation (between the acid and neutral structures of Cerulean), the chromophore cavity retains a very similar spatial organization, including the presence of several structural water molecules.

Figure S11 illustrates first the permanency, in all these structures, of a "water layer" formed by 3 to 5 water molecules lying in a plane parallel to the chromophore conjugated system. In GFPs and CFPs with a "Down" configuration, this water layer is composed of three well conserved water molecules (shown as grey spheres), while the water layer is more variable in the case of CFPs in the "Up" configuration. Each of these three major water molecules is H-bonded to at least one amino-acid whose role was found significant in the genetic engineering of GFPs, namely from right to left, on each of the structures shown in Figure S11:

- the first water molecule is the most well conserved of the layer. It is always bound to the main chain nitrogen of Val68, and to the residue 65 hydroxyl when it is in the "Down" configuration

- the 2nd water molecule is bound to Glu222 in all GFP and CFP structures, except in the acid structure of Cerulean (2Q57).

- a 3rd (or 4rth) water molecule is always bound to the amide nitrogen of the Gln69 side chain, and to the Thr203 hydroxyl.

The next finding illustrated by Figure S11 is the existence of two typical orientations of the residue 65 hydroxyl ("Down" and "Up"), that are adopted independently of the type of fluorescent protein (either GFP, CFP or Cerulean) :

- the "Down" configuration is characterized by a H-bond with the water layer (see above)

- the "Up" configuration is characterized by an alternate H-bonding to either the Val61 main chain carbonyl, or to the imidazolinone nitrogen.

In all cases ("Up" or "Down"), a H-bond of the residue 65 hydroxyl to Glu222 is found, but its configuration varies widely, owing to alternate binding possibilities on the two oxygens of the Glu222 caboxyl group, and the variable orientations adopted by this side chain.

REFERENCES

1. Malo GD, Pouwels LJ, Wang M, Weichsel A, Montfort WR, et al. (2007) X-ray structure of Cerulean GFP: a tryptophan-based chromophore useful for fluorescence lifetime imaging. Biochemistry 46: 9865-9873.

2. Sinnecker D, Voigt P, Hellwig N, Schaefer M (2005) Reversible photobleaching of enhanced green fluorescent proteins. Biochemistry 44: 7085-7094.

3. Markwardt ML, Kremers GJ, Kraft CA, Ray K, Cranfill PJ, et al. (2011) An improved cerulean fluorescent protein with enhanced brightness and reduced reversible photoswitching. PLoS One 6: e17896.

4. Merola F, Levy B, Demachy I, Pasquier H (2010) Photophysics and Spectroscopy of Fluorophores in the Green Fluorescent Protein Family. In: Demchenko AP, editor. Advanced Fluorescence Reporters in Chemistry and Biology" Vol I Fundamentals and Molecular Design Springer Series on Fluorescence: Springer. pp. 347-383.

5. Goedhart J, von Stetten D, Noirclerc-Savoye M, Lelimousin M, Joosen L, et al. (2012) Structure-guided evolution of cyan fluorescent proteins towards a quantum yield of 93%. Nat Commun 3: doi: 10.1038.
